# The impact of the National Syphilis Prevention Program on the prevalence of syphilis among people living with HIV in China: a systematic review and meta‐analysis

**DOI:** 10.1002/jia2.26408

**Published:** 2025-01-06

**Authors:** Qingling Zeng, Yuhui Yang, Limin Zhang, Jiangyu Yan, Jian Wang, Jingmin Nie, Qingmei Wang, Yu Luo, Gaoming Li

**Affiliations:** ^1^ Department of Cardiovascular Xinqiao Hospital Army Medical University Chongqing China; ^2^ School of Nursing Army Medical University Chongqing China; ^3^ Clinical Research Center Chongqing Public Health Medical Center Chongqing China; ^4^ Center for Disease Control and Prevention of Central Theater Command of Chinese People's Liberation Army Beijing China; ^5^ Department of Infectious Disease People's Hospital of Chongqing Banan District Chongqing China; ^6^ Department of Nursing, The First Medical Center Chinese PLA General Hospital Beijing China

**Keywords:** syphilis, HIV, meta‐analysis, coinfection, sexually transmitted infections/diseases, China

## Abstract

**Introduction:**

In 2010, China launched the 10‐year National Syphilis Prevention and Control Program to curb the spread of syphilis by integrating syphilis screening and treatment with HIV services. Herein, we aimed to evaluate changes in the prevalence of syphilis among people living with HIV (PLHIV) in China.

**Methods:**

We conducted this systematic review and meta‐analysis by searching the PubMed, Embase, Web of Science, China Biomedical Literature, China National Knowledge Infrastructure, Wanfang and CQVIP databases from inception to 1 June 2024 to obtain relevant articles. A total of 75 studies were ultimately included. We used a DerSimonian‒Laird random effects model to estimate the prevalence and 95% confidence interval of syphilis among PLHIV.

**Results:**

The overall prevalence of syphilis among PLHIV in China was 18.6% (95% CI 16.5–21.0). Regional differences (*R* ^2^ = 15.29%) were observed in the prevalence rates: 22.2% (18.9–25.8) in the eastern region, 19.0% (15.1–23.8) in the central region and 14.0% (11.1–17.5) in the western region. The prevalence decreased from 22.8% (18.4–27.9) before 2010 to 17.0% (14.6–19.6) in 2010 and thereafter (*R* ^2^ = 5.82%). Among PLHIV via homosexual transmission, the prevalence of syphilis was 24.9% (21.3–28.9), which significantly declined from 33.8% (27.5–40.8) to 21.4% (18.3–24.9) in 2010 and thereafter (*R* ^2^ = 22.35%). The prevalence of syphilis was significantly higher in men living with HIV than in women living with HIV (pooled odds ratio 1.67, 95% CI 1.29–2.15), with the highest prevalence in the eastern region (2.55, 95% CI 1.80–3.59).

**Discussion:**

The prevalence of syphilis among PLHIV, particularly in cases of homosexual transmission, has declined. There was a correlation between the prevalence of syphilis and regional economic conditions, with a greater burden in developed eastern coastal areas. Additionally, the risk of syphilis differed across sexes, with men living with HIV having a higher risk.

**Conclusions:**

There has been preliminary success in the control of syphilis among PLHIV, but there is still a long way to go to meet the WHO's 2030 syphilis prevention and control goal. Syphilis prevention measures should be integrated into broader health policies and development plans, particularly in high‐burden regions and populations.

## 1 INTRODUCTION

Syphilis is a chronic and complex systemic disease caused by Treponema pallidum infection. It can lead to skin and mucous membrane damage, as well as nervous system and cardiovascular system injury, which can be life‐threatening. Thirty‐three to fifty‐two percent of syphilis patients are asymptomatic, thereby creating additional challenges for the diagnosis and prevention of syphilis [1]. It is estimated that there were 6.3 million new syphilis cases worldwide in 2016 [2], with 438,000 cases reported in China [3]; therefore, China has a heavy burden of syphilis.

Due to the introduction of antiretroviral therapy, acquired immunodeficiency syndrome (AIDS) has shifted from a serious and fatal disease to a chronic, controllable disease [4]. In China, although the prevalence of human immunodeficiency virus (HIV) among the general population is low, the total number of people living with HIV (PLHIV) continues to increase [5, 6]. According to a report from the Chinese Center for Disease Control and Prevention, 577,000 people in China were diagnosed with HIV at the end of 2015 [7]. By the end of 2020, this number had increased to 1.053 million, and homosexual transmission accounted for 23.3% of these cases [8].

Syphilis and AIDS are major public health problems in China. These two diseases have overlapping routes of transmission and affected populations and thereby affect each other in many ways. Epidemiological studies have shown that syphilis one of the most common causes of genital ulcers, while ulcer‐related epithelial and mucosal damage significantly increases the risk of HIV acquisition [9]. Since 2000, the number of syphilis cases reported in China has increased at a rate of approximately 30% per year [10, 11]. In 2008, the number of syphilis cases in Guangdong Province alone was greater than that in the entire European Union during the same period [12]. To reverse the rising trend of this disease, the Ministry of Health of China launched the first national plan, the China Syphilis Prevention and Control Program (2010–2020), which gradually integrated syphilis screening and treatment at HIV counselling and testing sites [13]. In addition, the World Health Organization (WHO) has proposed four ambitious goals for the prevention and control of sexually transmitted infections, one of which is to reduce the global incidence of syphilis, a curable sexually transmitted disease, by 90% between 2018 and 2030 [14, 15].

Given the ongoing challenge of syphilis in high‐burden populations, such as PLHIV, understanding the impact of the National Syphilis Prevention Program is critical to shaping future public health strategies. However, no national‐level study has evaluated the efficacy of syphilis prevention and treatment among PLHIV in China. Previous studies have focused mainly on estimating the prevalence of syphilis in a limited region and may not fully represent the overall situation in China [16, 17, 18, 19]. This study aims to evaluate changes in the prevalence of syphilis among PLHIV in China, thus providing a foundation for future policy and public health initiatives in alignment with the global syphilis control agenda.

## 2 METHODS

The study was conducted in accordance with the Preferred Reporting Items for Systematic Reviews and Meta‐Analyses (PRISMA) statement (Appendix S1) [20] and was registered on PROSPERO (CRD42022357963).

### 2.1 Search strategy

The PubMed, Embase, Web of Science, China Biomedical Literature (CBM), China National Knowledge Infrastructure (CNKI), Wanfang and CQVIP databases were searched from inception to 1 June 2024, to identify articles examining the prevalence of syphilis among PLHIV. Free terms combined with subject terms (i.e. MeSH terms), such as syphilis, Treponema pallidum, HIV, AIDS, acquired immunodeficiency syndrome, coinfection, and China, were used to search the databases (Appendix S2). The retrieved papers were managed via EndNote (version 20), and duplicates were eliminated. A search was also conducted in Google Scholar for grey literature. All papers included in the study were reviewed.

### 2.2 Selection criteria

The inclusion criteria were as follows: (1) articles published in peer‐reviewed journals; and (2) research conducted in China that reported the prevalence of syphilis among PLHIV, including the number of syphilis cases and the total sample size. If the study reported data in the form of proportions, these data were used to estimate the prevalence of syphilis in the relevant population.

The exclusion criteria were as follows: (1) abstracts, reviews, editorials, commentaries, newsletters or brief communications; (2) articles with an insufficient sample size (for the expected prevalence of 30% [21], the required sample size was 323 for the absolute precision of 5% in estimating the prevalence with 95% confidence [22]); or (3) articles including PLHIV with at least one other infectious disease in addition to syphilis (this kind of research excluded PLHIV who have not experienced coinfection, resulting in a significant selection bias; therefore, the findings would not represent the general population of PLHIV).

### 2.3 Syphilis categorization

In this review, syphilis testing methods were categorized according to the findings of the serological tests reported in the selected publications. The diagnosis of syphilis typically involves the detection of both treponemal and non‐treponemal antibodies [23]. Accordingly, the infection types were classified into the following categories on the basis of combinations of serological test results [24]: (1) probable current syphilis infection (positive results for both treponemal and non‐treponemal tests); (2) possible current infection, unspecified (positive non‐treponemal test results, regardless of the result from the treponemal test if performed); (3) lifetime syphilis infection (positive treponemal test results, regardless of the result from the non‐treponemal test if conducted); and (4) unclear (when the diagnostic method used was either not reported or insufficiently described).

### 2.4 Data extraction and bias risk assessment

The following data were extracted independently by two researchers (Zeng, Yang) via a unified data table: (1) baseline data of the included studies (first author, year of publication, study region, study population and syphilis testing method); (2) HIV acquisition status (the number of PLHIV and the composition by sex and transmission route); and (3) syphilis status (the number of syphilis infections and their composition in terms of sex and transmission route). If multiple studies used the same dataset, only the study with the highest quality and the largest sample size or the most detailed information was selected. Disagreements between the two reviewers were resolved via discussion and consensus. If an agreement could not be reached, a third reviewer was consulted.

The Joanna Briggs Institute critical appraisal checklist (Appendix S3) was used [25] to evaluate the quality of all included studies. The checklist consists of nine items divided into three aspects: study participants (Items 1, 2, 4 and 9), outcome measurement (Items 6 and 7) and statistical analysis (Items 3, 5 and 8). By answering yes, no, unclear or not applicable to each question, bias in the literature can be identified. A document that satisfies the criteria of a given item is counted as 1 point. Two independent reviewers assessed the quality of the studies, and their scores were averaged together to yield a total score, which ranged from 0 to 9. A score less than 6 was defined as high risk, a score of 6–7 was defined as medium risk and a score of 8–9 was defined as low risk. The kappa test was used to analyse the inter‐rater reliability of each item in the checklist, and Spearman's rank correlation was used to analyse the total scores for the included studies.

### 2.5 Data analysis

All the statistical analyses were performed via R4.1.3. All the statistical tests were two‐tailed, and *p* < 0.05 was considered statistically significant unless otherwise stated. Logit transformation was used to stabilize the variance in prevalence. A DerSimonian‒Laird random effects model was used to estimate pooled prevalence rates and 95% confidence intervals (CIs). For publications that reported the prevalence of syphilis among PLHIV of different sexes, we also used a random effects model to calculate the combined odds ratio (OR) and its 95% CI between the two groups [26]. The Cochrane *Q* test was used to analyse the heterogeneity among the studies. The statistic *Q* approximately followed a *χ* ^2^ distribution with *k*‐1 degrees of freedom (*k* is the number of studies). Heterogeneity between studies was indicated at *p* < 0.10. Additionally, the magnitude of heterogeneity was quantitatively evaluated on the basis of Higgins's *I* ^2^ value, which ranges from 0% to 100% [27]. An *I* ^2^ greater than 50% generally indicates greater heterogeneity. A funnel plot was used to analyse whether the included literature had potential publication bias, and Egger's linear regression method was used to test the asymmetry of the funnel plot [28].

Subgroup analyses were performed to explore how study characteristics may influence the reported prevalence of syphilis among PLHIV. The differences in prevalence between studies were explored through univariate meta‐regression. In the meta‐regression analysis, the dependent variable was the prevalence of syphilis or the effect size of the comparison of the prevalence of different sexes. The independent variables were region (dummy variable: Eastern region), study time (dummy variable: Before 2010), population source (dummy variable: Community‐based), risk of bias (dummy variable: High) and sample size (dummy variable: <1000 patients). In the meta‐regression analysis, the restricted maximum likelihood method was used to estimate the variance between studies, and the proportion of variance explained by any meta^−^regression model was estimated via the *R* ^2^ statistic [29].

## 3 RESULTS

### 3.1 Literature overview

**Figure 1:**
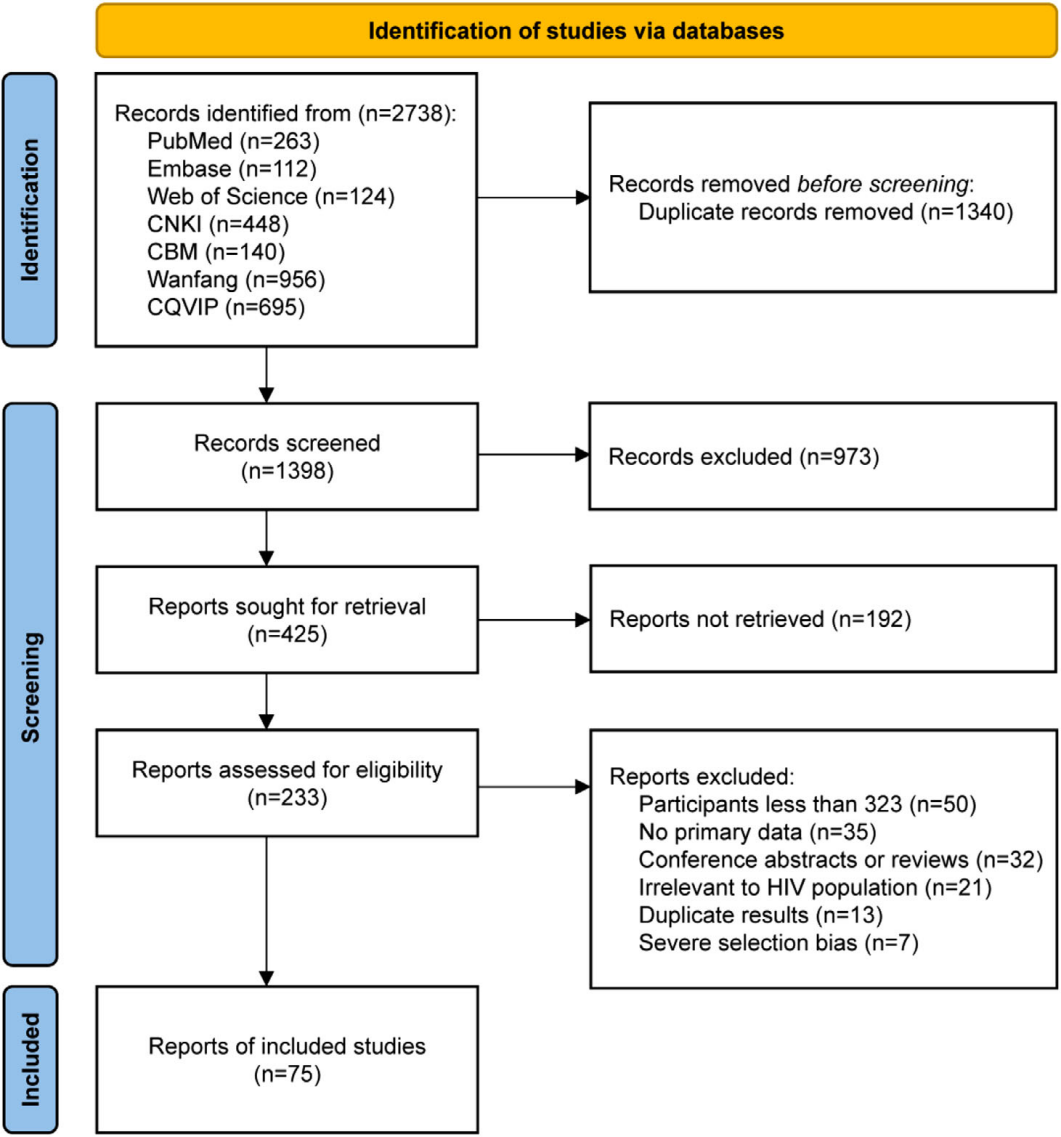
Literature inclusion and exclusion process.

The literature search initially yielded 2738 relevant studies. After excluding duplicates and conducting preliminary screening of the titles and abstracts, we comprehensively examined 233 papers, of which 50 had a sample size of less than 323 patients and 35 provided incomplete information; thus, complete data could not be obtained. There were 32 abstracts and reviews, 21 papers unrelated to PLHIV, 13 duplicate papers and 7 papers with severe selection bias. Ultimately, 75 papers met the eligibility criteria and were included for analysis (Figure 1).

### 3.2 Characteristics of the study participants

**Figure 2:**
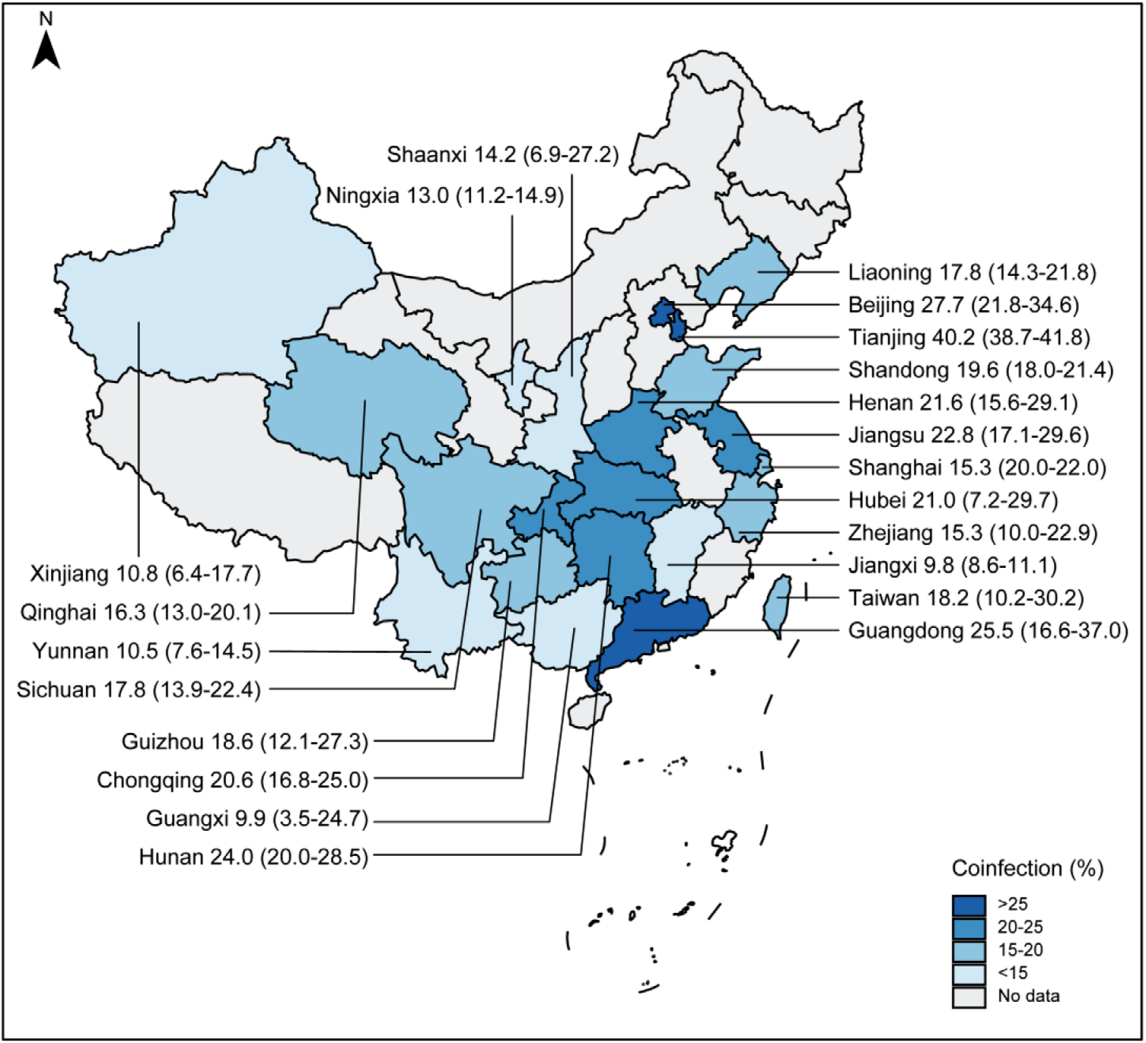
Geographic distribution of prevalence of syphilis among PLHIV in China.

The characteristics of the participants in the 75 eligible studies are shown in Table 1. There were 33, 17 and 14 studies that reported the number of syphilis cases among PLHIV via homosexual transmission, heterosexual transmission and intravenous drug use, respectively. A total of 138,324 PLHIV were included across all the eligible studies, and the number of patients in each study ranged from 325 to 15,123. These studies were conducted between 2000 and 2023 and were conducted in 22 of the 34 provinces in China (Figure 2). Among these studies, 37 were conducted in the eastern coastal region, 8 were conducted in the central region, 28 were conducted in the western region and 2 were conducted in multiple provinces. Most of the studies were based on community population surveys (45 of 75).

**Table 1:** Main characteristics of the included studies

	Study characteristics				Prevalence of syphilis coinfection (syphilis cases/total samples)						
	Year	Region	Population	Syphilis testing method	Overall	Homosexual	Heterosexual	Intravenous drug use	Male	Female	Risk of bias
Lu et al. [30]	2006–2009	Western region	Community‐based	RPR+ELISA	108/472	108/472	··	··	··	··	Low
Wang et al. [31]	2003–2007	Eastern region	Hospital‐based	RPR	67/330	··	··	··	··	··	Moderate
Chen et al. [32]	2008–2010	Eastern region	Community‐based	ELISA	191/678	··	··	··	··	··	Moderate
Zhao et al. [33]	2000–2010	Eastern region	Community‐based	RPR	409/2087	69/230	179/873	65/405	253/1311	156/776	Low
Zhu et al. [34]	2008–2010	Eastern region	Community‐based	ELISA	142/719	··	··	··	··	··	High
Cai et al. [35]	2005–2011	Eastern region	Community‐based	TRUST+TPPA	186/338	186/338	··	··	··	··	High
Fu et al. [36]	2009–2011	Western region	Hospital‐based	NR	150/843	··	··	··	··	··	Moderate
Tang and Yang [37]	2012–2013	Western region	Hospital‐based	ELISA	69/406	··	··	··	47/288	22/118	Low
Wang et al. [38]	2009–2012	Eastern region	Hospital‐based	TRUST+TPPA	150/686	··	··	··	··	··	Moderate
Wu et al. [21]	2008–2009	Multiple regions	Community‐based	RPR/TRUST+TPPA	693/2314	693/2314	··	··	··	··	Low
Hu et al. [39]	2009–2013	Eastern region	Hospital‐based	RPR+TPPA	200/1010	171/778	16/146	··	196/942	4/68	Low
Wang et al. [40]	2009–2013	Western region	Community‐based	ELISA+TPHA	78/595	··	··	78/595	31/426	47/169	Moderate
Wu et al. [16]	2013	Eastern region	Hospital‐based	RPR	179/920	··	··	··	··	··	Moderate
Yang and Yu [41]	2010–2013	Western region	Community‐based	TPPA	184/1087	··	··	5/41	143/792	41/295	Low
Chen et al. [42]	2000–2010	Eastern region	Community‐based	NR	1830/15,123	··	··	··	··	··	High
Guan et al. [43]	2008–2013	Eastern region	Community‐based	RPR+TPPA	175/448	175/448	··	··	··	··	High
Jia et al. [44]	2007–2012	Eastern region	Community‐based	RPR+TPPA	98/440	98/440	··	··	··	··	Moderate
Lu et al. [45]	2013–2014	Western region	Community‐based	WB	177/695	··	··	··	119/455	58/240	Moderate
Tong et al. [46]	2015	Western region	Hospital‐based	ELISA+TPPA	84/520	··	··	··	··	··	Low
Wu et al. [47]	2011–2012	Eastern region	Community‐based	RPR+TPPA	59/429	59/429	··	··	··	··	Moderate
Li et al. [48]	2013–2014	Eastern region	Hospital‐based	RPR+TPPA	408/1070	368/1122	25/109	··	··	··	High
Lu et al. [49]	2013–2015	Western region	Community‐based	WB	415/1742	··	27/153	12/19	254/1136	135/606	Moderate
Ma et al. [50]	2011–2014	Eastern region	Community‐based	TRUST+TPPA	264/1653	264/1653	··	··	··	··	Moderate
Ya et al. [51]	2010–2015	Eastern region	Community‐based	RPR+TPPA	51/421	51/421	··	··	··	··	High
Chen et al. [52]	2010–2016	Eastern region	Community‐based	ELISA	92/401	··	··	··	··	··	Low
Li [53]	2011–2015	Central region	Community‐based	ELISA+TPPA	122/463	29/69	84/309	4/38	91/302	31/161	Low
Lyu et al. [54]	2012–2016	Western region	Hospital‐based	TPPA	52/364	··	··	··	··	··	Moderate
Tsai et al. [55]	2000–2010	Eastern region	Community‐based	NR	3302/13,316	··	··	··	··	··	High
Xu et al. [56]	2014–2015	Eastern region	Hospital‐based	TRUST	213/870	··	··	··	210/838	3/32	Moderate
Yang et al. [57]	2014–2016	Western region	Hospital‐based	CLIA+TPPA	169/894	28/80	114/667	2/6	137/668	32/226	Low
Yu et al. [58]	2002–2014	Western region	Community‐based	TRUST+ELISA	160/1235	··	··	··	136/1051	24/184	Low
Yuan et al. [59]	2015	Eastern region	Community‐based	RPR+TPPA	197/2090	··	··	··	152/1264	45/826	Low
Cao et al. [60]	2011–2016	Central region	Hospital‐based	CMIA	156/709	··	··	··	··	··	Moderate
Chen et al. [61]	2016–2017	Eastern region	Community‐based	ELISA	72/564	··	··	··	··	··	Low
Lan et al. [62]	2013–2015	Western region	Community‐based	RPR+ELISA	107/486	107/486	··	··	··	··	Moderate
Li et al. [63]	2010–2015	Eastern region	Community‐based	NR	72/460	··	··	··	··	··	Moderate
Liu et al. [64]	2017	Eastern region	Community‐based	RPR+TPPA	111/385	111/385	··	··	··	··	Moderate
Shi [65]	2014–2016	Central region	Community‐based	ELISA	168/673	··	··	··	··	··	Moderate
Zhao et al. [66]	2011–2016	Western region	Community‐based	RPR+TPHA	80/581	32/112	43/363	4/90	67/406	13/175	Low
Chen et al. [67]	2003–2018	Central region	Hospital‐based	RPR+TPPA	178/1870	··	··	··	··	··	Low
Gao et al. [68]	2010–2017	Eastern region	Community‐based	RPR+TPPA	89/639	89/639	··	··	··	··	High
Wang et al. [69]	2013–2016	Western region	Community‐based	TRUST+TPPA	21/325	21/325	··	··	··	··	Moderate
Weng et al. [70]	2009–2017	Eastern region	Community‐based	TRUST+TPPA	293/561	293/561	··	··	··	··	Moderate
Wu et al. [71]	2013–2017	Eastern region	Community‐based	RPR+RT	655/3514	53/236	··	41/142	621/3139	34/375	Low
Zhu et al. [72]	2013–2017	Eastern region	Community‐based	RPR+TPPA	46/331	46/331	··	··	··	··	Low
Li et al. [73]	2009–2015	Western region	Community‐based	TRUST+TPPA	214/2162	··	··	··	··	··	Moderate
Ling and Hu [74]	2012–2019	Western region	Hospital‐based	NR	153/543	··	··	··	··	··	Moderate
Mamatiaili et al. [75]	2004–2016	Western region	Hospital‐based	TRUST+TPPA	31/381	··	··	··	··	··	Moderate
Sun et al. [76]	2015–2018	Eastern region	Hospital‐based	RPR+TPPA	886/4493	698/3154	172/1218	··	872/4160	14/333	Moderate
Yang et al. [77]	2018	Western region	Hospital‐based	ELISA	53/496	··	··	··	32/323	21/173	Low
Yi et al. [78]	2011–2018	Western region	Hospital‐based	RPR/TRUST+ELISA	325/11,045	··	··	··	··	··	Moderate
Zhang et al. [79]	2008–2018	Eastern region	Hospital‐based	TRUST+ELISA	159/370	··	··	··	··	··	Moderate
Zhang et al. [17]	2011–2016	Central region	Community‐based	ELISA	265/1018	95/256	155/667	11/55	237/835	28/183	Low
Zhu et al. [80]	2014–2018	Eastern region	Community‐based	TRUST+ELISA	184/1205	56/248	120/825	5/92	145/942	39/263	Low
Chen et al. [81]	2013–2018	Eastern region	Community‐based	TPPA	197/618	197/618	··	··	··	··	Moderate
Cheng et al. [82]	2019–2020	Eastern region	Hospital‐based	RPR+TPPA	111/527	··	··	··	108/474	3/53	Low
Fan et al. [83]	2009–2019	Eastern region	Hospital‐based	RPR	1539/3829	1063/2501	241/716	··	1511/3637	28/192	Moderate
Li et al. [84]	2020	Western region	Community‐based	TRUST+ELISA	344/5922	58/292	283/5469	3/161	254/4107	90/1815	Moderate
Liu and Kang [85]	2018–2020	Western region	Hospital‐based	TRUST+ELISA	71/476	··	··	··	··	··	Moderate
Wang et al. [86]	2013–2017	Central region	Hospital‐based	ELISA+TPPA	65/359	··	··	··	··	··	Low
Zhang et al. [87]	2019–2020	Central region	Hospital‐based	ELISA	46/426	··	··	··	36/277	10/149	Moderate
Zhang et al. [18]	2009–2014	Multiple regions	Community‐based	RPR+TPPA	119/400	119/400	··	··	··	··	Low
He and Wu [88]	2012–2021	Western region	Hospital‐based	NR	264/1164	··	··	··	··	··	High
Jin et al. [89]	2017–2020	Eastern region	Community‐based	NR	191/1961	115/721	76/1229	··	··	··	Low
Tu et al. [19]	2020–2021	Western region	Hospital‐based	TRUST+TPPA	81/406	··	··	··	··	··	Low
Zheng et al. [90]	2013–2019	Eastern region	Hospital‐based	NR	1038/9840	··	··	··	971/8403	67/1437	Low
Liu et al. [91]	2019–2021	Eastern region	Hospital‐based	TPPA	120/401	··	··	··	··	··	Moderate
Pan et al. [92]	2020–2021	Western region	Community‐based	TRUST+ELISA	136/1858	25/122	104/1664	7/72	110/1369	26/489	Low
Qin et al. [93]	2019–2021	Western region	Community‐based	RPR+TPPA	333/2869	··	··	··	··	··	Moderate
Shan et al. [94]	2021–2022	Eastern region	Hospital‐based	TRUST+ELISA	125/389	76/224	49/165	··	118/342	7/47	Low
Xu et al. [95]	2020–2021	Eastern region	Community‐based	RPR+RT	520/1679	··	··	··	489/1500	31/179	Low
You et al. [96]	2017–2020	Western region	Community‐based	NR	68/418	··	··	··	··	··	Moderate
Zhang et al. [97]	2013–2021	Western region	Community‐based	NR	698/8636	285/2065	314/5624	0/662	··	··	Moderate
Yang et al. [98]	2010–2020	Central region	Hospital‐based	RPR	1391/6623	··	··	··	··	··	Low
Yang et al. [99]	2023	Western region	Community‐based	TRUST+ELISA	445/2053	26/126	416/1903	3/24	333/1432	112/621	Low

Abbreviations: CLIA, chemiluminescence immunoassay; CMIA, chemiluminescent microparticle immunoassay; ELISA, enzyme‐linked immunosorbent assay; NR, not reported; RPR, rapid plasma reagin; RT, rapid test; TPHA, Treponema pallidum haemagglutination assay; TPPA, Treponema pallidum particle agglutination; TRUST, toluidine red unheated serum test; WB, western blotting.

### 3.3 Risk of bias in studies

The results of the risk of bias assessment indicated that the two evaluators had a high degree of inter‐rater reliability; the average kappa coefficient of the items in the risk of bias assessment list was 0.85 (range: 0.72–1.00; Appendix S4). Spearman's rank correlation coefficient (*r*) of the total score of the studies was 0.75 (*p* < 0.001). The scores for the 75 studies included in the analysis ranged from 4 to 9 points, with an average score of 7.2 ± 1.1. There were 31 low‐risk studies, 35 medium‐risk studies and 9 high‐risk studies (Appendix S5).

### 3.4 Overall prevalence

**Figure 3:**
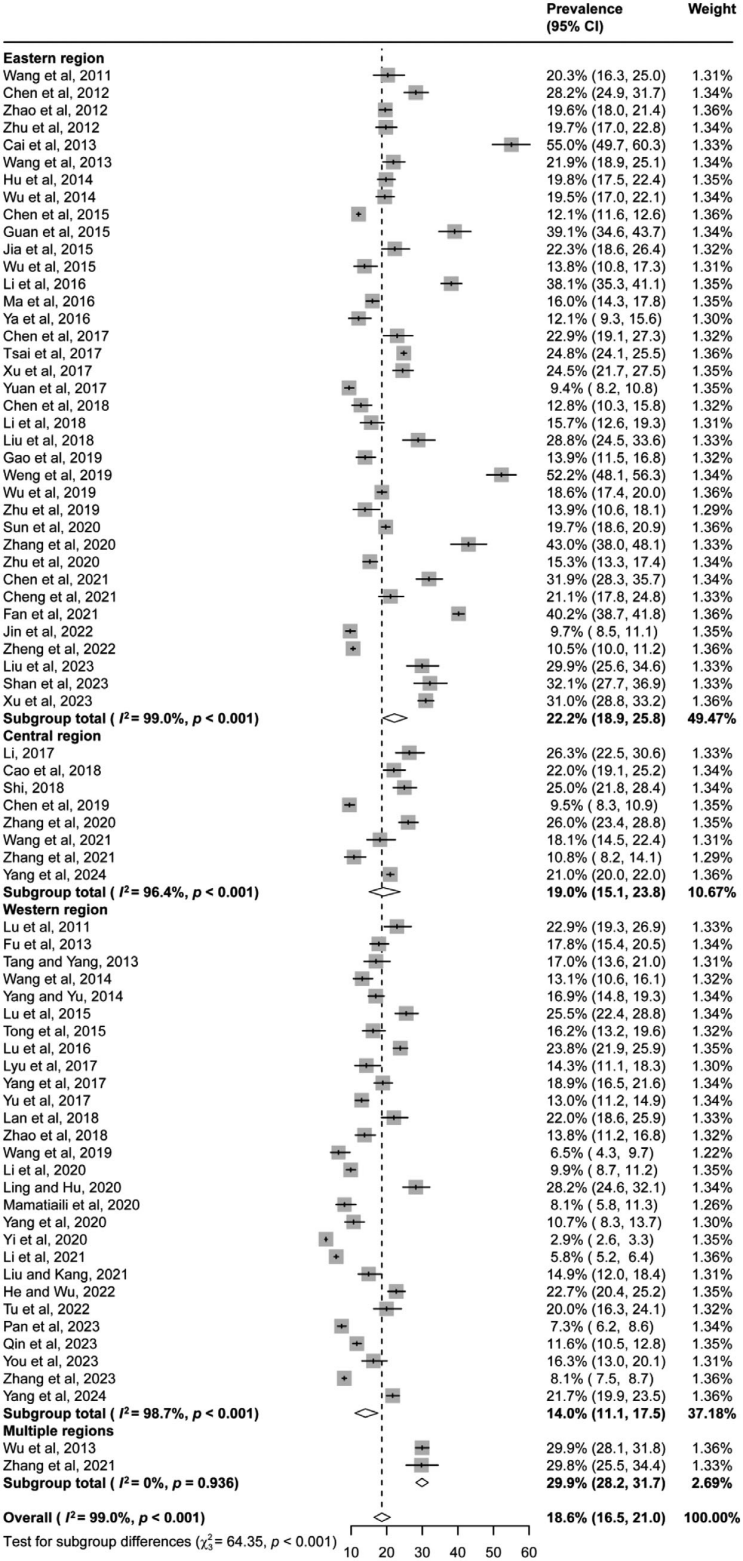
Forest plots of prevalence of syphilis among PLHIV in China. CI, confidence interval.

**Figure 4:**
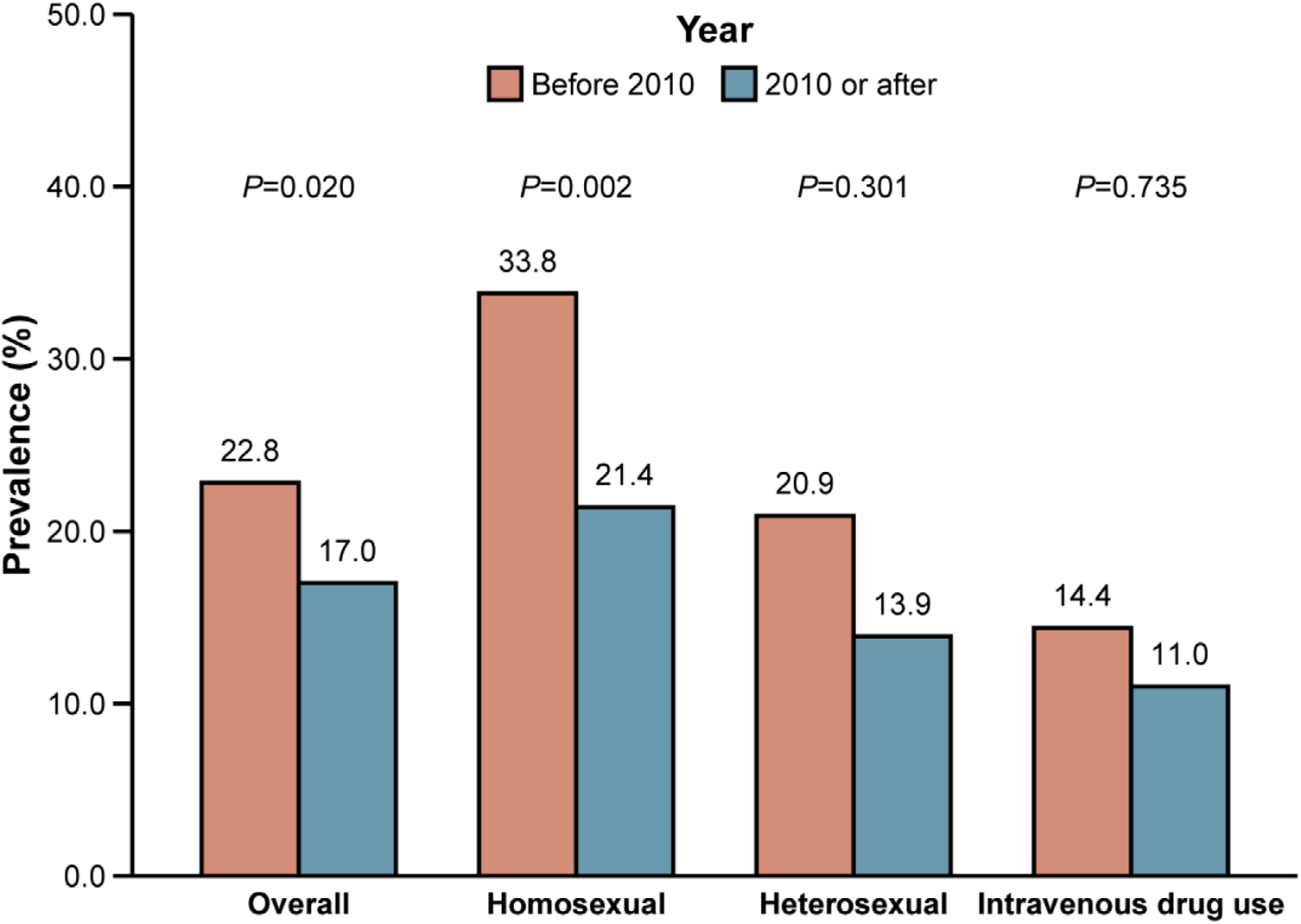
Prevalence of syphilis among PLHIV by transmission route.

The prevalence of syphilis in the included studies ranged from 2.9% (95% CI 2.6–3.3) to 55.0% (49.7–60.3) (Figure 3). There was significant heterogeneity among studies, with a Higgins’ *I* ^2^ value of 99.0 (*Q* test *p* < 0.001). The overall prevalence of syphilis among PLHIV was 18.6% (16.5–21.0; Table 2). Specifically, the prevalence was 22.2% (18.9–25.8) in the eastern region, where the prevalence in three provinces exceeded 25%, that is 40.2% (38.7–41.8) in Tianjin, 27.7% (21.8–34.6) in Beijing and 25.5% (16.6–37.0; Figure 2) in Guangdong. The prevalence was 19.0% (15.1–23.8) in the central region, and the prevalence was 14.0% (11.1–17.5) in the western region. Studies reported that the pooled prevalence of syphilis in PLHIV was 22.8% (18.4–27.9) before 2010 and 17.0% (14.6–19.6) after 2010 (Figure 4). Univariate meta‐regression analysis was performed using the random effects model and revealed that region (*R* ^2^ = 15.29%), study time (*R* ^2^ = 5.82%) and sample size (*R* ^2^ = 5.40%) might be potential sources of heterogeneity; in contrast, no significant difference was found in terms of population (*R* ^2^ < 0.01%), risk of bias (*R* ^2^ = 0.98%) or syphilis testing method (*R* ^2^ < 0.01%). Egger's test revealed that there was no evidence of publication bias in the overall prevalence of syphilis among PLHIV (*t* = −0.077, *p* = 0.939; Appendix Figure S1).

**Table 2:** Pooled prevalence of syphilis among PLHIV determined via a random effects model

	Subgroup analysis					Univariate meta‐regression			
	Number of studies	Number of individuals with syphilis coinfection	Number of PLHIV	Prevalence of syphilis coinfection (95% CI)	*I* ^2^ (%)	β (SE)	OR (95% CI)	*p* value	*R* ^2^ (%)
Region									
Eastern region	37	14,621	74,795	22.2% (18.9–25.8)	99.0	Ref	Ref	Ref	15.29
Central region	8	2391	12,141	19.0% (15.1–23.8)	96.4	—0.194 (0.233)	0.823 (0.522–1.300)	0.405	
Western region	28	5070	48,674	14.0% (11.1–17.5)	98.7	—0.558 (0.150)	0.573 (0.427–0.768)	<0.001	
Multiple regions	2	812	2714	29.9% (28.2–31.7)	0.0	0.402 (0.432)	1.495 (0.641–3.487)	0.352	
Year									
Before 2010	23	10,472	50,207	22.8% (18.4–27.9)	99.2	Ref	Ref	Ref	5.82
2010 or after	52	12,422	88,117	17.0% (14.6–19.6)	98.7	—0.368 (0.158)	0.692 (0.508–0.943)	0.020	
Population									
Community‐based	45	14,361	86,064	18.5% (16.0–21.4)	98.9	Ref	Ref	Ref	<0.01
Hospital‐based	30	8533	52,260	18.8% (14.9–23.3)	99.2	0.016 (0.154)	1.016 (0.752–1.374)	0.916	
Risk of bias									
High	9	6447	33,238	24.3 (17.4–32.8)	99.4	Ref	Ref	Ref	1.21
Moderate	35	8205	56,433	18.2 (14.3–23.0)	99.3	—0.361 (0.240)	0.697 (0.435–1.116)	0.133	
Low	31	8242	48,653	17.6 (15.2–20.3)	98.0	—0.407 (0.243)	0.666 (0.413–1.073)	0.095	
Sample size									
<1000 patients	46	5226	23,856	20.7% (18.3–23.4)	95.9	Ref	Ref	Ref	5.40
≥1000 patients	29	17,668	114,468	15.8% (12.8–19.4)	99.5	—0.331 (0.149)	0.718 (0.536–0.962)	0.027	
Syphilis testing method									
Probable current syphilis infection	38	8413	57,533	18.1 (14.6–22.3)	99.1	Ref	Ref	Ref	<0.01
Possible current infection, unspecified	6	3798	14,659	23.7 (16.8–32.3)	99.1	0.333 (0.284)	1.395 (0.800–2.433)	0.241	
Lifetime syphilis infection	21	2917	13,828	19.9 (17.6–22.5)	92.4	0.107 (0.177)	1.113 (0.787–1.573)	0.546	
Unclear infection type	10	7766	52,304	15.5 (11.3–21.0)	99.5	—0.186 (0.230)	0.830 (0.529–1.302)	0.417	
Total	75	22,894	138,324	18.6% (16.5–21.0)	99.0				

Abbreviations: CI, confidence interval; OR, odds ratio; PLHIV, people living with HIV; Ref, reference category; SE, standard error.

### 3.5 Prevalence by transmission route

We further analysed the prevalence of syphilis among PLHIV based on different transmission routes (Appendices S6–S8): 24.9% (21.3–28.9) of cases involved homosexual transmission, 14.9% (10.6–20.6) involved heterosexual transmission and 12.4% (8.3–18.3) involved intravenous drug use. The results of univariate meta‐regression analysis indicated that the study year (*R* ^2^ = 22.35%) might be a potential source of heterogeneity in the homosexual transmission subgroup. Compared with the results (33.8% [27.5–40.8]) of studies conducted before 2010, the prevalence of syphilis among PLHIV was significantly lower in studies conducted after 2010 (21.4% [18.3–24.9]) (Figure 4).

### 3.6 Effects of sex

**Figure 5:**
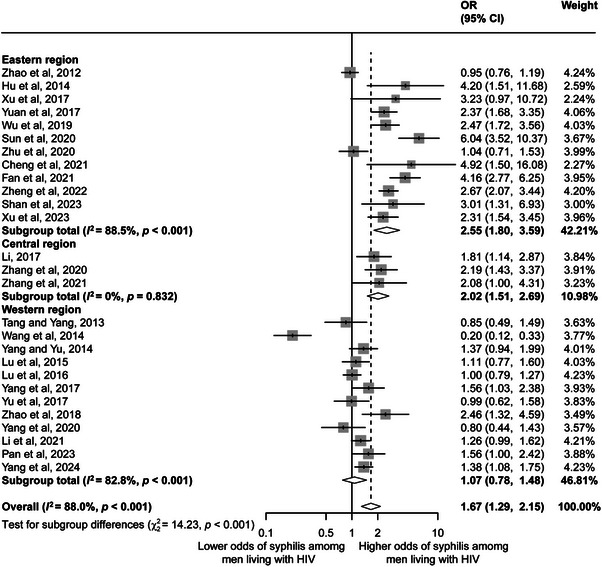
Forest plot of prevalence of syphilis among men living with HIV compared with women living with HIV in China. CI, confidence interval; OR, odds ratio.

Twenty‐seven studies included in the meta‐analysis were used to compare the prevalence of syphilis among PLHIV between different sexes. Compared with women living with HIV, men living with HIV were 1.67 times more likely to develop syphilis (95% CI 1.29–2.15; Figure 5). The three covariates of region (*R* ^2^ = 35.13%), population source (*R* ^2^ = 15.84%) and syphilis testing method (*R* ^2^ = 12.41%) were statistically significant in the univariate meta‐regression analysis (Table 3). The risk of syphilis among males was 2.55 times greater than that among females (95% CI 1.80–3.59) in the eastern region and 2.02 (95% CI 1.51–2.69) times greater than that among females in the central region; the difference in the western region was not significant (1.07 [0.78–1.48]). According to the subgroup analysis of the population source, the risk of syphilis in men was greater than that in women in both the hospital and community populations, with odds ratios of 2.44 (95% CI 1.60–3.71) and 1.34 (95% CI 1.01–1.78), respectively. In addition, the prevalence was higher among men than among women in the probable current syphilis infection group (2.05 [95% CI 1.52–2.77]).

**Table 3:** Pooled odds ratios for sex and the risk of syphilis among PLHIV determined via a random effects model

	Subgroup analysis					Univariate meta‐regression			
	Number of studies	Number of men living with HIV	Number of women living with HIV	OR (95% CI)	*I* ^2^ (%)	β (SE)	OR (95% CI)	*p* value	*R* ^2^ (%)
Region									
Eastern region	12	26,952	4581	2.55 (1.80–3.59)	88.5	Ref	Ref	Ref	35.13
Central region	3	1414	493	2.02 (1.51–2.69)	0.0	—0.228 (0.372)	0.796 (0.384–1.652)	0.541	
Western region	12	12,453	5111	1.07 (0.78–1.48)	82.8	—0.858 (0.232)	0.424 (0.269–0.667)	<0.00	
Year									
Before 2010	5	7367	1389	1.24 (0.42–3.70)	95.7	Ref	Ref	Ref	3.45
2010 or after	22	33,452	8796	1.76 (1.43–2.16)	79.8	0.418 (0.333)	1.519 (0.791–2.919)	0.209	
Population									
Community‐based	16	20,467	7357	1.34 (1.01–1.78)	86.7	Ref	Ref	Ref	15.84
Hospital‐based	11	20,352	2828	2.44 (1.60–3.71)	81.2	0.595 (0.254)	1.813 (1.102–2.981)	0.019	
Risk of bias									
Moderate	8	15,036	3536	1.58 (0.75–3.33)	94.4	Ref	Ref	Ref	<0.01
Low	19	25,783	6649	1.68 (1.37–2.05)	78.8	—0.110 (0.293)	0.896 (0.505–1.590)	0.707	
Sample size									
<1000 patients	11	4799	1543	1.42 (0.85–2.38)	87.0	Ref	Ref	Ref	2.52
≥1000 patients	16	36,020	8642	1.84 (1.40–2.40)	88.4	0.291 (0.268)	1.337 (0.790–2.263)	0.279	
Syphilis testing method									
Probable current syphilis infection	13	21,128	5428	2.05 (1.52–2.77)	80.1	Ref	Ref	Ref	12.41
Possible current infection, unspecified	3	5786	1000	2.22 (0.82–5.98)	95.1	0.027 (0.424)	1.027 (0.448–2.357)	0.949	
Lifetime syphilis infection	10	5502	2320	1.10 (0.72–1.68)	86.8	—0.638 (0.269)	0.528 (0.312–0.896)	0.018	
Unclear infection type	1	8403	1437	2.67 (2.07–3.44)	NA	0.246 (0.626)	1.279 (0.375–4.364)	0.694	
Total	27	40,819	10,185	1.67 (1.29–2.15)	88.0				

Abbreviations: CI, confidence interval; OR, odds ratio; Ref, reference category; SE, standard error.

## 4 DISCUSSION

To our knowledge, this is the first systematic review and meta‐analysis to assess the prevalence and burden of syphilis among PLHIV after the implementation of the National Syphilis Prevention and Treatment Plan in China. This systematic review summarized the data from 75 studies that were conducted across 22 provinces and included a total of 138,324 participants. Three main findings were observed. First, since the implementation of the National Syphilis Prevention and Treatment Plan, the prevalence of syphilis among PLHIV has shown a decreasing trend. This trend was consistent across different transmission routes but was more obvious for homosexual transmission, with a decrease of approximately one‐third. Second, there was a correlation between the prevalence of syphilis among PLHIV and the regional economy, such that the disease burden was greater in developed coastal areas in the eastern region. Third, there were differences in the risk of syphilis among different sexes in this study; the overall risk of syphilis was greater among men, and it was 2.55 times greater than that among women in the eastern region.

In the 1960s, syphilis almost disappeared from the public eye and the medical community in China [100]. In the late 1970s, China implemented economic reforms, and the social environment and norms underwent major changes, leading to changes in sexual attitudes and sexual behaviours, the migration of the population from rural or underdeveloped areas to cities, the privatization of the healthcare system and insufficient investment in public health infrastructure [101]. These changes created conditions that were conducive to the resurgence of syphilis. Owing to a lack of international support, a lack of awareness of the epidemic and solutions by decision‐makers, and the general negative stigma of syphilis, the status of syphilis in the national policy agenda is low [102, 103]. Since the early 2000s, the spread of syphilis has been curbed mainly through health education and the promotion of condom use [104]. This behavioural change‐based intervention did not achieve the expected results. The incidence of syphilis reported in 2010 was more than three times greater than that reported in 2000 [3]. Given the trend of the continuous spread of syphilis in China, the government has realized the necessity of strengthening the response to syphilis and thus launched the National Syphilis Prevention and Treatment Plan [13].

From an epidemiological perspective, syphilis is closely associated with HIV acquisition. Combining syphilis prevention and HIV control programmes, expanding the coverage of testing and increasing the syphilis detection rate in key populations are critical approaches for the early detection and treatment of syphilis patients, thereby reducing transmission [105]. The rapid uptake of HIV testing, including self‐testing, has made it easier for high‐risk populations to access HIV diagnosis and care [106, 107]. A robust HIV control system infrastructure has laid the foundation for expanding syphilis testing in PLHIV [108]. Syphilis screening and treatment services have gradually been integrated into HIV counselling and testing sites, and routine syphilis testing is carried out for this population. These measures provide a solid foundation for syphilis control in the country [102]. The results of this study revealed that the prevalence of syphilis among PLHIV in China decreased from 22.8% before 2010 to 17.0% after 2010 and that the decrease in the prevalence rate of syphilis among the homosexual transmission population was even more significant, from 33.8% to 21.4%. Nevertheless, the total prevalence of 24.9% in the homosexual transmission population of PLHIV is much higher than that in the general men who have sex with men (MSM) population (9.7%) [109]. The finding that the prevalence of syphilis is greater via homosexual transmission is consistent with previous research, which showed that MSM living with HIV are at greater risk for syphilis due to sexual behaviours that increase exposure to syphilis and other sexually transmitted infections [110]. Additionally, the significant decline in the prevalence of syphilis within this key population after 2010 is promising and is likely attributable to targeted public health interventions, such as focused syphilis testing and treatment programmes designed for MSM [105, 111]. The integration of these programmes with HIV prevention and treatment services has become a cornerstone of syphilis control strategies in China. However, despite these improvements, MSM in China continue to face substantial stigma and discrimination [112]. This social marginalization creates significant barriers to accessing health services, including syphilis testing and treatment [113]. Studies have shown that many MSM avoid seeking healthcare due to fear of being outed or mistreated, leading to under‐reporting of syphilis and HIV status [114]. This stigma not only impedes access to critical health services but also contributes to delays in diagnosis and treatment, which can exacerbate the spread of syphilis within this population and to the general public [115]. Efforts to address discrimination are essential to ensure that successful syphilis reduction is sustainable in the long term.

The prevalence of syphilis among PLHIV exhibits regional differences. The syphilis burden among PLHIV was the highest in the eastern region of the country (22.2%), that is 3.2% and 8.2% higher than those in the central and western regions of China, respectively. This finding is also consistent with the distribution of the syphilis population released by the infectious disease surveillance system in China [11]. In addition, the prevalence of syphilis differed across sexes. The prevalence of syphilis was 2.55 times higher among men living with HIV than among women living with HIV in the eastern region and 2.02 times higher in the central region; no difference between males and females was observed in the western region. The higher syphilis rates observed in the eastern region can be partly attributed to the rapid development of the commercial sex industry and the concentration of high‐risk populations in economically developed urban areas [116]. Urbanization has led to a greater number of MSM, migrant workers and sex workers, all of whom are at greater risk for syphilis. Sex tourism also plays an important role in the prevalence of regional sexually transmitted diseases, and syphilis is the most common diagnosis among travellers and immigrants [117, 118]. The eastern region, particularly large cities such as Beijing, Shanghai and Guangzhou, attract many migrant workers and are the most popular destinations for sex tourism. In a study in Beijing, 40% of MSM who responded had more than 10 lifetime male sex partners [119]. While the eastern region has higher rates of syphilis, this may also reflect better healthcare infrastructure and more widespread testing, leading to greater detection and treatment. In contrast, the central and western regions, which are less developed economically, have more limited healthcare resources [3]. This results in lower testing and treatment coverage, contributing to the persistence of syphilis in these regions. The relative under‐reporting and underdiagnosis in the central and western regions may also contribute to the lower observed prevalence in these regions than in the east. In addition, changes in population structure may also lead to regional differences in prevalence and risk between sexes, which have changed the structure and scale of the traditional high‐risk population [120]. The sex ratio of the Chinese population is severely imbalanced, and demographers estimate that there is a surplus of more than 32 million unmarried men younger than 20 [121]. Most of these men are unable to find employment due to poverty and a low level of education, leading to their migration from rural areas to economically developed cities. A model study on sex [120, 122] indicated that these excess male sexual demands may increase the risk of sexually transmitted diseases and expand the demand for commercial sexual behaviour.

Increasing the coverage or frequency of syphilis testing for key populations can reduce the prevalence of syphilis [123]. The WHO guidelines recommend that key populations in sexually active populations should be tested for syphilis at least once per year and that those who are at higher risk and those who use preexposure prophylaxis should be tested once every 3 months [124, 125]. Since pregnant women bear the most serious burden of adverse consequences, since 2010, prenatal HIV and syphilis intervention has been included as part of routine prenatal care in China. By 2013, 96.4% of pregnant women in areas covered by the integrated programme had undergone HIV and syphilis testing [126]. However, only 30% of the MSM population has been tested for syphilis, which is far lower than the 69% of the MSM population that has been tested for syphilis in the United States [127, 128]. Approximately half of the female sex workers in China still do not know their syphilis status [129]. Stigma and discrimination make many people reluctant to seek voluntary counselling and testing services. Confidentiality and privacy are the most important factors that affect a participant's decision to test and where to test [130]. The emergence of new technologies provides the possibility to further improve the efficiency and expand the coverage of testing. For example, a rapid dual HIV/syphilis test kit provides additional opportunities for contacting individuals who have not been tested before. This type of kit is inexpensive, sensitive and specific [131]. A previous meta‐analysis revealed that the results of the rapid dual HIV/syphilis test were comparable to the results of the laboratory reference test [132]. Currently, the WHO has prequalified three different rapid dual HIV/syphilis tests and recommends rapid dual diagnostic tests as the first test for prenatal care [133]. In addition, during the COVID‐19 pandemic, the diagnosis rates of HIV and syphilis decreased due to social distancing and lockdown measures, making it difficult for high‐risk populations to undergo testing and obtain treatment [134]. The use of rapid dual HIV/syphilis testing tools to meet the testing needs of local communities worldwide during the pandemic is critical for the prevention of HIV and syphilis [135].

Although the prevalence of syphilis among PLHIV has significantly decreased in China over the past 10 years, PLHIV still disproportionately bear the burden of syphilis. There is still a long way to go to achieve the goal of a 90% reduction in the global incidence of syphilis by 2030. To further reduce syphilis prevalence, China should expand syphilis screening and testing, especially in high‐risk populations such as MSM and sex workers, and incorporate rapid dual HIV/syphilis testing, particularly in underserved areas. Addressing the barriers posed by stigma and discrimination in key populations, such as MSM, will improve healthcare access and testing uptake. Strengthening regional surveillance systems, especially in central and western China, is crucial for targeted interventions. Socio‐economic disparities should be addressed by improving healthcare access in rural areas through outreach services and telemedicine consultations [136, 137]. Additionally, aligning national policies with the WHO's 2030 goals, including expanding maternal syphilis elimination programmes and integrating sexual health services, will help achieve the global target of a 90% reduction in syphilis incidence.

Although this study was conducted in strict accordance with the PRISMA guidelines, the following limitations still need to be considered. First, half (37/75) of the data surveys and studies were conducted in the eastern coastal area of China, and the combined prevalence rate in this area was 22.2%, indicating that syphilis monitoring among PLHIV has attracted more attention and is more common in economically developed coastal areas. Furthermore, this finding indicated that the estimate of the prevalence of syphilis in China may be slightly greater. Second, most of the studies were retrospective studies with non‐probability sampling, and the subjects of 16 studies were male homosexual patients. Therefore, the estimated prevalence cannot be representative of all PLHIV in China. Third, the prevalence data of some provinces and cities are missing. Small‐sample studies are prone to accidental and inaccurate results, and such studies were excluded from the analysis. Excluding these studies may result in a meta‐analysis that fails to fully reflect publication bias. More high‐quality contemporary data from more provinces and cities are critical for estimating and understanding the prevalence of syphilis among PLHIV. Fourth, some individuals who were identified as living with HIV via intravenous drug use may also engage in sexual risk behaviours that could increase the likelihood of syphilis acquisition; without a clear stratification of sexual risk behaviour, the prevalence of syphilis in the intravenous drug use group may be overestimated. Fifth, our classification of syphilis on the basis of serological tests has limitations. Individuals with a history of adequately treated syphilis may still test positive on non‐treponemal tests for years. Without antibody titre cut‐offs, some cases classified as probable current syphilis infections may actually reflect past infections, leading to potential overestimation of the current syphilis prevalence.

## 5 CONCLUSIONS

China is one of the few countries with a National Syphilis Prevention and Treatment Plan. Through the strategic integration of syphilis screening and treatment with HIV services, preliminary positive results have been achieved. This integration can be used as a reference and can be replicated in other countries, especially those with a high burden of syphilis. In the future, China needs to develop priority policies for syphilis interventions in key areas and key populations to promote comprehensive screening, treatment and prevention services for syphilis in high‐risk populations.

## COMPETING INTERESTS

The authors declare that they have no competing interests.

## AUTHORS’ CONTRIBUTIONS

GL, QZ and YL conceived and designed the study. QZ and YY performed the literature search. QZ and LZ worked on data extraction and collection. GL, JY and JW analysed and interpreted the data. QZ, YY and JN wrote the initial draft of the report. GL, YL and QW critically revised subsequent versions of the report. All the authors reviewed and approved the final report.

## FUNDING

This project was supported by the Center for Disease Control and Prevention of Central Theater Command (jkzx2022‐02) and the Chongqing Municipal Health Commission (2021jstg038).

## Supporting information




**Appendix S1**. The PRISMA statement.
**Appendix S2**. Search strategy used in each database.
**Appendix S3**. Joanna Briggs Institute critical appraisal checklist for studies reporting prevalence data.
**Appendix S4**. Consistency test of the items in the bias risk assessment checklist.
**Appendix S5**. Evaluation results of the included literature.
**Appendix S6**. Prevalence of syphilis among PLHIV via homosexual transmission.
**Appendix S7**. Prevalence of syphilis among PLHIV via the heterosexual transmission route.
**Appendix S8**. Prevalence of syphilis among PLHIV via intravenous drug use.
**Appendix Figure S1**. Funnel plots and Egger's tests for the outcomes: (A) overall, (B) homosexual transmission, (C) heterosexual transmission, (D) intravenous drug use, and (E) sex comparison.

## Data Availability

All raw data from the 75 analysed studies are available in the main text and Supporting Information; further inquiries can be directed to the corresponding author.
