# Supplementary material for: The impact of the National Syphilis Prevention Program on the prevalence of syphilis among people living with HIV in China: a systematic review and meta‐analysis
Source: J Int AIDS Soc. 2025 Jan 6;28(1):e26408. doi: 10.1002/jia2.26408 (PMC11705538; doi:10.1002/jia2.26408)
Supplement: Supplementary file 1 — Appendix S1. The PRISMA statement. Appendix S2. Search strategy used in each database. Appendix S3. Joanna Briggs Institute critical appraisal checklist for studies reporting prevalence data. Appendix S4. Consistency test of the items in the bias risk assessment checklist. Appendix S5. Evaluation results of the included literature. Appendix S6. Prevalence of syphilis among PLHIV via homosexual transmission. Appendix S7. Prevalence of syphilis among PLHIV via the heterosexual transmission route. Appendix S8. Prevalence of syphilis among PLHIV via intravenous drug use. Appendix Figure S1. Funnel plots and Egger's tests for the outcomes: (A) overall, (B) homosexual transmission, (C) heterosexual transmission, (D) intravenous drug use, and (E) sex comparison. [file JIA2-28-e26408-s001.docx]

**SUPPORTING INFORMATION**

**Table of contents**

[Appendix S1. The PRISMA statement. 2](#_Toc31902)

[Appendix S2. Search strategy used in each database. 11](#_Toc9060)

[Appendix S3. Joanna Briggs Institute critical appraisal checklist for studies reporting prevalence data. 12](#_Toc6035)

[Appendix S4. Consistency test of the items in the bias risk assessment checklist. 13](#_Toc29170)

[Appendix S5. Evaluation results of the included literature. 13](#_Toc11458)

[Appendix S6. Prevalence of syphilis among PLHIV via homosexual transmission. 15](#_Toc4733)

[Appendix S7. Prevalence of syphilis among PLHIV via the heterosexual transmission route. 16](#_Toc762)

[Appendix S8. Prevalence of syphilis among PLHIV via intravenous drug use. 17](#_Toc24552)

[Appendix Figure S1. Funnel plots and Egger’s tests for the outcomes: (A) overall, (B) homosexual transmission, (C) heterosexual transmission, (D) intravenous drug use, and (E) sex comparison. 18](#_Toc17308)

**Appendix S1. The PRISMA statement.**

| **Section and Topic** | **Item #** | **Checklist item** | **Location where item is reported** |
| --- | --- | --- | --- |
| **TITLE** | | |  |
| Title | 1 | Identify the report as a systematic review. | Title:  “The Impact of the National Syphilis Prevention Program on the Prevalence of Syphilis among People Living with HIV in China: A Systematic Review and Meta-analysis” |
| **ABSTRACT** | | |  |
| Abstract | 2 | See the PRISMA 2020 for Abstracts checklist. | Abstract:  “In 2010, China launched the 10-year National Syphilis Prevention and Control Program to curb the spread of syphilis by integrating syphilis screening and treatment with HIV services. Herein, we aimed to evaluate changes in the prevalence of syphilis among people living with HIV (PLHIV) in China. (…)” |
| **INTRODUCTION** | | |  |
| Rationale | 3 | Describe the rationale for the review in the context of existing knowledge. | Introduction, paragraph 4:  “Given the ongoing challenge of syphilis in high-burden populations, such as PLHIV, understanding the impact of the National Syphilis Prevention Program is critical to shaping future public health strategies. However, no national-level study has evaluated the efficacy of syphilis prevention and treatment among PLHIV in China. Previous studies have focused mainly on estimating the prevalence of syphilis in a limited region and may not fully represent the overall situation in China (…)” |
| Objectives | 4 | Provide an explicit statement of the objective(s) or question(s) the review addresses. | Introduction, paragraph 4:  This study aims to evaluate changes in the prevalence of syphilis among PLHIV in China, thus providing a foundation for future policy and public health initiatives in alignment with the global syphilis control agenda.” |
| **METHODS** | | |  |
| Eligibility criteria | 5 | Specify the inclusion and exclusion criteria for the review and how studies were grouped for the syntheses. | Methods, Selection criteria:  “The inclusion criteria were as follows: (…)”  “The exclusion criteria were as follows: (…)” |
| Information sources | 6 | Specify all databases, registers, websites, organisations, reference lists and other sources searched or consulted to identify studies. Specify the date when each source was last searched or consulted. | Methods, paragraph 2:  “The PubMed, Embase, Web of Science, China Biomedical Literature (CBM), China National Knowledge Infrastructure (CNKI), Wanfang and CQVIP databases were searched from inception to June 1, 2024, to identify articles examining the prevalence of syphilis among PLHIV.” |
| Search strategy | 7 | Present the full search strategies for all databases, registers and websites, including any filters and limits used. | Methods, paragraph 2:  “Free terms combined with subject terms (i.e., MeSH terms), such as syphilis, Treponema pallidum, HIV, AIDS, acquired immunodeficiency syndrome, coinfection, and China, were used to search the databases (Appendix S2).” |
| Selection process | 8 | Specify the methods used to decide whether a study met the inclusion criteria of the review, including how many reviewers screened each record and each report retrieved, whether they worked independently, and if applicable, details of automation tools used in the process. | Methods, paragraph 2:  “The retrieved papers were managed via EndNote (version 20), and duplicates were eliminated. A search was also conducted in Google Scholar for grey literature. All papers included in the study were reviewed.” |
| Data collection process | 9 | Specify the methods used to collect data from reports, including how many reviewers collected data from each report, whether they worked independently, any processes for obtaining or confirming data from study investigators, and if applicable, details of automation tools used in the process. | Methods, paragraph 6:  “The following data were extracted independently by two researchers (Zeng, Yang) via a unified data table: (…)” |
| Data items | 10a | List and define all outcomes for which data were sought. Specify whether all results that were compatible with each outcome domain in each study were sought (e.g. for all measures, time points, analyses), and if not, the methods used to decide which results to collect. | Methods, paragraph 6:  “(1) baseline data of the included studies (first author, year of publication, study region, study population, and syphilis testing method); (2) HIV acquisition status (the number of PLHIV and the composition by sex and infection route); and (3) syphilis infection status (the number of syphilis infections and their composition in terms of sex and route of infection).” |
|  | 10b | List and define all other variables for which data were sought (e.g. participant and intervention characteristics, funding sources). Describe any assumptions made about any missing or unclear information. | Methods, paragraph 6:  “(1) baseline data of the included studies (first author, year of publication, study region, study population, and syphilis testing method); (2) HIV acquisition status (the number of PLHIV and the composition by sex and infection route); and (3) syphilis infection status (the number of syphilis infections and their composition in terms of sex and route of infection).” |
| Study risk of bias assessment | 11 | Specify the methods used to assess risk of bias in the included studies, including details of the tool(s) used, how many reviewers assessed each study and whether they worked independently, and if applicable, details of automation tools used in the process. | Methods, paragraph 7:  “The Joanna Briggs Institute critical appraisal checklist (Appendix S3) was used [25] to evaluate the quality of all included studies (…)” |
| Effect measures | 12 | Specify for each outcome the effect measure(s) (e.g. risk ratio, mean difference) used in the synthesis or presentation of results. | Methods, paragraph 8:  “A DerSimonian‒Laird random effects model was used to estimate pooled prevalence rates and 95% confidence intervals (CIs). For publications that reported the prevalence of syphilis among PLHIV of different sexes, we also used a random effects model to calculate the combined odds ratio (OR) and its 95% CI between the two groups [26].” |
| Synthesis methods | 13a | Describe the processes used to decide which studies were eligible for each synthesis (e.g. tabulating the study intervention characteristics and comparing against the planned groups for each synthesis (item #5)). | Methods, paragraph 8:  “A DerSimonian‒Laird random effects model was used to estimate pooled prevalence rates and 95% confidence intervals (CIs). For publications that reported the prevalence of syphilis among PLHIV of different sexes, we also used a random effects model to calculate the combined odds ratio (OR) and its 95% CI between the two groups [26].” |
|  | 13b | Describe any methods required to prepare the data for presentation or synthesis, such as handling of missing summary statistics, or data conversions. | Methods, paragraph 8:  “Logit transformation was used to stabilize the variance in prevalence.” |
|  | 13c | Describe any methods used to tabulate or visually display results of individual studies and syntheses. | Methods, paragraph 8:  “A funnel plot was used to analyse whether the included literature had potential publication bias, and Egger's linear regression method was used to test the asymmetry of the funnel plot [28].” |
|  | 13d | Describe any methods used to synthesize results and provide a rationale for the choice(s). If meta-analysis was performed, describe the model(s), method(s) to identify the presence and extent of statistical heterogeneity, and software package(s) used. | Methods, paragraph 8:  “The Cochrane *Q* test was used to analyse the heterogeneity among the studies. The statistic *Q* approximately followed a *χ^2^* distribution with *k* -1 degrees of freedom (*k* is the number of studies). Heterogeneity between studies was indicated at *P* was less than 0.10. Additionally, the magnitude of heterogeneity was quantitatively evaluated based on Higgins's *I*^2^ value, which ranges from 0% to 100%[27]. An *I*^2^ greater than 50% generally indicates greater heterogeneity.” |
|  | 13e | Describe any methods used to explore possible causes of heterogeneity among study results (e.g. subgroup analysis, meta-regression). | Methods, paragraph 9:  “Subgroup analyses were performed to explore how study characteristics may influence the reported prevalence of syphilis among PLHIV. The differences in prevalence between studies were explored through univariate meta-regression. In the meta-regression analysis, the dependent variable was the prevalence of syphilis or the effect size of the comparison of the prevalence of different sexes (…)” |
|  | 13f | Describe any sensitivity analyses conducted to assess robustness of the synthesized results. | NA |
| Reporting bias assessment | 14 | Describe any methods used to assess risk of bias due to missing results in a synthesis (arising from reporting biases). | Methods, paragraph 8:  “A funnel plot was used to analyse whether the included literature had potential publication bias, and Egger's linear regression method was used to test the asymmetry of the funnel plot [28].” |
| Certainty assessment | 15 | Describe any methods used to assess certainty (or confidence) in the body of evidence for an outcome. | Methods, paragraph 9:  “In the meta-regression analysis, the restricted maximum likelihood method was used to estimate the variance between studies, and the proportion of variance explained by any meta-regression model was estimated via the R2 statistic [29].” |
| **RESULTS** | | |  |
| Study selection | 16a | Describe the results of the search and selection process, from the number of records identified in the search to the number of studies included in the review, ideally using a flow diagram. | Results, paragraph 1, Figure1:  “The literature search initially yielded 2738 relevant studies (…)” |
|  | 16b | Cite studies that might appear to meet the inclusion criteria, but which were excluded, and explain why they were excluded. | Results, paragraph 1, Figure1:  “of which 50 had a sample size of less than 323 patients, and 35 provided incomplete information and thus complete data could not be obtained (…)” |
| Study characteristics | 17 | Cite each included study and present its characteristics. | Results, paragraph 2, Table 1:  “The characteristics of the participants in the 75 eligible studies are shown in Table 1 (…)” |
| Risk of bias in studies | 18 | Present assessments of risk of bias for each included study. | Results, paragraph 3, Supporting Information:  “The results of the risk of bias assessment indicated that the two evaluators had a high degree of interrater reliability (…)” |
| Results of individual studies | 19 | For all outcomes, present, for each study: (a) summary statistics for each group (where appropriate) and (b) an effect estimate and its precision (e.g. confidence/credible interval), ideally using structured tables or plots. | Results, paragraph 4-6, Supporting Information:  See Figure 2-5. |
| Results of syntheses | 20a | For each synthesis, briefly summarise the characteristics and risk of bias among contributing studies. | Results, paragraph 4-6, Table 2-3, Figure 2-5, Supporting Information. |
|  | 20b | Present results of all statistical syntheses conducted. If meta-analysis was done, present for each the summary estimate and its precision (e.g. confidence/credible interval) and measures of statistical heterogeneity. If comparing groups, describe the direction of the effect. | Results, paragraph 4-6, Figure 2-5:  See Table 2-3 and Supporting Information. |
|  | 20c | Present results of all investigations of possible causes of heterogeneity among study results. | Results, paragraph 4-6, Table 2-3, Figure 2-5, Supporting Information:  “Univariate meta-regression analysis was performed using the random effects model and revealed that region (*R*^2^ = 15.29%), study time (*R*^2^ = 5.82%), and sample size (*R*^2^ = 5.40%) might be potential sources of heterogeneity (…)”  “The results of univariate meta-regression analysis indicated that study year (*R*^2^ = 22.35%) might be a potential source of heterogeneity in the homosexual transmission subgroup”  “The 3 covariates of region (*R*^2^ = 35.13%), population source (*R*^2^ = 15.84%) and syphilis testing method (*R*^2^ = 12.41%) were statistically significant in the univariate meta-regression analysis (Table 3)” |
|  | 20d | Present results of all sensitivity analyses conducted to assess the robustness of the synthesized results. | NA |
| Reporting biases | 21 | Present assessments of risk of bias due to missing results (arising from reporting biases) for each synthesis assessed. | Results, paragraph 4-6:  See Table 2-3 and Supporting Information. |
| Certainty of evidence | 22 | Present assessments of certainty (or confidence) in the body of evidence for each outcome assessed. | Results, paragraph 4-6:  See Table 2-3 and Supporting Information. |
| **DISCUSSION** | | |  |
| Discussion | 23a | Provide a general interpretation of the results in the context of other evidence. | Discussion, paragraphs 1:  “First, since the implementation of the National Syphilis Prevention and Treatment Plan, the prevalence of syphilis among PLHIV has shown a decreasing trend (…)”: |
|  | 23b | Discuss any limitations of the evidence included in the review. | Discussion, paragraphs 7:  “Although this study was conducted in strict accordance with the PRISMA guidelines, the following limitations still need to be considered (…)” |
|  | 23c | Discuss any limitations of the review processes used. | Discussion, paragraphs 7:  “Although this study strictly followed the PRISMA guidelines, the following limitations still need to be considered (…)” |
|  | 23d | Discuss implications of the results for practice, policy, and future research. | Discussion, paragraphs 8:  “This integration can be used as a reference and can be replicated in other countries, especially those with a high burden of syphilis (…)” |
| **OTHER INFORMATION** | | |  |
| Registration and protocol | 24a | Provide registration information for the review, including register name and registration number, or state that the review was not registered. | Methods, paragraphs 1:  “and was registered on PROSPERO (CRD42022357963) before the meta-analysis was conducted.” |
|  | 24b | Indicate where the review protocol can be accessed, or state that a protocol was not prepared. | Methods, paragraphs 1:  “and was registered on PROSPERO (CRD42022357963) before the meta-analysis was conducted.” |
|  | 24c | Describe and explain any amendments to information provided at registration or in the protocol. | NA |
| Support | 25 | Describe sources of financial or non-financial support for the review, and the role of the funders or sponsors in the review. | See “FUNDING” Section |
| Competing interests | 26 | Declare any competing interests of review authors. | See “COMPETING INTERESTS” Section |
| Availability of data, code and other materials | 27 | Report which of the following are publicly available and where they can be found: template data collection forms; data extracted from included studies; data used for all analyses; analytic code; any other materials used in the review. | See “DATA AVAILABILITY STATEMENT” Section |

**Appendix S2. Search strategy used in each database.**

PubMed

| Step | Query | Results |
| --- | --- | --- |
| 1 | (("HIV"[Mesh]) OR ("Acquired Immunodeficiency Syndrome"[Mesh])) OR ((((HIV) OR (Human Immunodeficiency Virus)) OR (AIDS)) OR (Acquired Immune Deficiency Syndrome)) | 567170 |
| 2 | ((("Syphilis"[Mesh]) OR ("Treponema pallidum"[Mesh])) OR (Syphilis)) OR (Treponema pallidum) | 43080 |
| 3 | ("Coinfection"[Mesh]) OR ((((Coinfection*) OR (Co-infection*)) OR (Co infection*)) OR (Mixed Infection*)) | 652678 |
| 4 | ("China"[Mesh]) OR ((China) OR (Chinese)) | 3287599 |
| 5 | Step 1 AND Step 2 AND Step 3 AND Step 4 | 263 |

Embase

| Step | Query | Results |
| --- | --- | --- |
| 1 | 'human immunodeficiency virus'/exp OR 'acquired immune deficiency syndrome'/exp OR hiv:ti,ab,kw OR 'human immunodeficiency virus':ti,ab,kw OR aids:ti,ab,kw OR 'acquired immune deficiency syndrome':ti,ab,kw | 1041936 |
| 2 | 'syphilis'/exp OR 'treponema pallidum'/exp OR syphilis:ti,ab,kw OR 'treponema pallidum':ti,ab,kw | 58278 |
| 3 | 'coinfection'/exp OR coinfection*:ti,ab,kw OR 'co infection*':ti,ab,kw OR 'mixed infection*':ti,ab,kw | 87763 |
| 4 | 'china'/exp OR china:ti,ab,kw OR chinese:ti,ab,kw | 751778 |
| 5 | Step 1 AND Step 2 AND Step 3 AND Step 4 | 112 |

Web of Science

| Step | Query | Results |
| --- | --- | --- |
| 1 | (((ALL=(HIV)) OR ALL=("Human Immunodeficiency Virus")) OR ALL=(AIDS)) OR ALL=("Acquired Immune Deficiency Syndrome") | 886459 |
| 2 | (ALL=(Syphilis)) OR ALL=(Treponema pallidum) | 15281 |
| 3 | (((ALL=(Coinfection*)) OR ALL=(Co-infection*)) OR ALL=(Co infection*)) OR ALL=(Mixed Infection*) | 154452 |
| 4 | (ALL=(China)) OR ALL=(Chinese) | 7784535 |
| 5 | Step 1 AND Step 2 AND Step 3 AND Step 4 | 124 |

CNKI

| Step | Query | Results |
| --- | --- | --- |
| 1 | (SU % 'HIV'+'Human Immunodeficiency Virus'+'AIDS'+'Acquired Immune Deficiency Syndrome'+'艾滋病'+‘获得性免疫缺陷综合症’) AND (SU % 'Syphilis'+'Treponema pallidum'+'梅毒') AND (SU % 'Coinfection'+'Co-infection'+'Co infection'+'Mixed Infection'+'合并感染')  限定：中文文献 | 448 |

CBM

| Step | Query | Results |
| --- | --- | --- |
| 1 | "HIV"[不加权:扩展] OR "获得性免疫缺陷综合征"[不加权:扩展] OR "HIV"[常用字段:智能] OR "Human Immunodeficiency Virus"[常用字段:智能] OR "AIDS"[常用字段:智能] OR "Acquired Immune Deficiency Syndrome"[常用字段:智能] OR "艾滋病"[常用字段:智能] OR "获得性免疫缺陷综合症"[常用字段:智能] | 79137 |
| 2 | "苍白密螺旋体"[不加权:扩展] OR "Syphilis"[常用字段:智能] OR "梅毒"[常用字段:智能] OR "Treponema pallidum"[常用字段:智能] | 23081 |
| 3 | "Coinfection"[常用字段:智能] OR "Co-infection"[常用字段:智能] OR "Co infection"[常用字段:智能] OR "Mixed Infection"[常用字段:智能] OR"合并感染"[常用字段:智能] | 19256 |
| 4 | Step 1 AND Step 2 AND Step 3 | 140 |

Wanfang

| Step | Query | Results |
| --- | --- | --- |
| 1 | (((((((主题=HIV) OR 主题=("Human Immunodeficiency Virus")) OR 主题=AIDS) OR 主题=("Acquired Immune Deficiency Syndrome")))) OR 主题=艾滋病) OR 主题=获得性免疫缺陷综合症 | 101881 |
| 2 | (主题=Syphilis) OR (主题=Treponema pallidum) OR 主题=梅毒 | 27109 |
| 3 | ((((主题=Coinfection) OR 主题="Co-infection") OR 主题=("Co infection")) OR 主题=("Mixed Infection")) OR 主题=合并感染 | 72980 |
| 4 | Step 1 AND Step 2 AND Step 3  限定：中文文献 | 956 |

CQVIP

| Step | Query | Results |
| --- | --- | --- |
| 1 | ((((U=HIV OR U="Human Immunodeficiency Virus") OR U=AIDS) OR U="Acquired Immune Deficiency Syndrome") OR (U=艾滋病 OR U=获得性免疫缺陷综合症)) | 303180 |
| 2 | (U=Syphilis OR U=Treponema pallidum OR U=梅毒) | 23293 |
| 3 | ((((U=Coinfection OR U="Co-infection") OR U="Co infection") OR U="Mixed Infection") OR U=合并感染) | 57239 |
| 4 | Step 1 AND Step 2 AND Step 3 | 695 |

**Appendix S3. Joanna Briggs Institute critical appraisal checklist for studies reporting prevalence data.**

| Criteria | Yes | No | Unclear | Not applicable |
| --- | --- | --- | --- | --- |
| 1. Was the sample frame appropriate to address the target population? |  |  |  |  |
| 2. Were study participants sampled in an appropriate way? |  |  |  |  |
| 3. Was the sample size adequate (at least 323 participants)? |  |  |  |  |
| 4. Were the study subjects and the setting described in detail? |  |  |  |  |
| 5. Was the data analysis conducted with sufficient coverage of the identified sample? |  |  |  |  |
| 6. Were valid methods used for the identification of the condition? |  |  |  |  |
| 7. Was the condition measured in a standard, reliable way for all participants? |  |  |  |  |
| 8. Was there appropriate statistical analysis? |  |  |  |  |
| 9. Was the response rate adequate, and if not, was the low response rate managed appropriately? |  |  |  |  |

**Appendix S4. Consistency test of the items in the bias risk assessment checklist.**

| Item Number | Kappa | Agreement |
| --- | --- | --- |
| 1 | 0.715 | 86.67% |
| 2 | 0.874 | 97.33% |
| 3 | 1.000 | 100.00% |
| 4 | 0.860 | 97.33% |
| 5 | 0.747 | 96.00% |
| 6 | 0.723 | 93.33% |
| 7 | 0.850 | 98.67% |
| 8 | 0.762 | 93.33% |
| 9 | 1.000 | 100.00% |
| **Mean** | **0.837** | **95.85%** |

**Appendix S5. Evaluation results of the included literature.**

| Study | Reviewer 1 | Reviewer 2 | Mean | Risk of bias |
| --- | --- | --- | --- | --- |
| Lu et al., 2011 | 8 | 8 | 8 | Low |
| Wang et al., 2011 | 7 | 8 | 7.5 | Moderate |
| Chen et al., 2012 | 6 | 6 | 6 | Moderate |
| Zhao et al., 2012 | 8 | 8 | 8 | Low |
| Zhu et al., 2012 | 5 | 5 | 5 | High |
| Cai et al., 2013 | 5 | 6 | 5.5 | High |
| Fu et al., 2013 | 6 | 6 | 6 | Moderate |
| Tang and Yang, 2013 | 8 | 8 | 8 | Low |
| Wang et al., 2013 | 7 | 7 | 7 | Moderate |
| Wu et al., 2013 | 8 | 8 | 8 | Low |
| Hu et al., 2014 | 9 | 8 | 8.5 | Low |
| Wang et al., 2014 | 7 | 7 | 7 | Moderate |
| Wu et al., 2014 | 6 | 6 | 6 | Moderate |
| Yang and Yu, 2014 | 8 | 8 | 8 | Low |
| Chen et al., 2015 | 4 | 4 | 4 | High |
| Guan et al., 2015 | 5 | 5 | 5 | High |
| Jia et al., 2015 | 7 | 7 | 7 | Moderate |
| Lu et al., 2015 | 6 | 7 | 6.5 | Moderate |
| Tong et al., 2015 | 8 | 8 | 8 | Low |
| Wu et al., 2015 | 7 | 7 | 7 | Moderate |
| Li et al., 2016 | 6 | 5 | 5.5 | High |
| Lu et al., 2016 | 8 | 7 | 7.5 | Moderate |
| Ma et al., 2016 | 7 | 7 | 7 | Moderate |
| Ya et al., 2016 | 4 | 4 | 4 | High |
| Chen et al., 2017 | 8 | 8 | 8 | Low |
| Li, 2017 | 8 | 8 | 8 | Low |
| Lyu et al., 2017 | 6 | 6 | 6 | Moderate |
| Tsai et al., 2017 | 5 | 5 | 5 | High |
| Xu et al., 2017 | 8 | 7 | 7.5 | Moderate |
| Yang et al., 2017 | 8 | 8 | 8 | Low |
| Yu et al., 2017 | 8 | 8 | 8 | Low |
| Yuan et al., 2017 | 8 | 8 | 8 | Low |
| Cao et al., 2018 | 7 | 8 | 7.5 | Moderate |
| Chen et al., 2018 | 8 | 8 | 8 | Low |
| Lan et al., 2018 | 7 | 7 | 7 | Moderate |
| Li et al., 2018 | 7 | 7 | 7 | Moderate |
| Liu et al., 2018 | 7 | 7 | 7 | Moderate |
| Shi, 2018 | 7 | 7 | 7 | Moderate |
| Zhao et al., 2018 | 8 | 8 | 8 | Low |
| Chen et al., 2019 | 8 | 8 | 8 | Low |
| Gao et al., 2019 | 5 | 4 | 4.5 | High |
| Wang et al., 2019 | 6 | 8 | 7 | Moderate |
| Weng et al., 2019 | 8 | 7 | 7.5 | Moderate |
| Wu et al., 2019 | 8 | 8 | 8 | Low |
| Zhu et al., 2019 | 8 | 8 | 8 | Low |
| Li et al., 2020 | 8 | 7 | 7.5 | Moderate |
| Ling and Hu, 2020 | 7 | 7 | 7 | Moderate |
| Mamatiaili et al., 2020 | 8 | 7 | 7.5 | Moderate |
| Sun et al., 2020 | 8 | 7 | 7.5 | Moderate |
| Yang et al., 2020 | 8 | 8 | 8 | Low |
| Yi et al., 2020 | 8 | 7 | 7.5 | Moderate |
| Zhang et al., 2020 | 7 | 8 | 7.5 | Moderate |
| Zhang et al., 2020 | 8 | 8 | 8 | Low |
| Zhu et al., 2020 | 8 | 8 | 8 | Low |
| Chen et al., 2021 | 5 | 7 | 6 | Moderate |
| Cheng et al., 2021 | 8 | 8 | 8 | Low |
| Fan et al., 2021 | 7 | 8 | 7.5 | Moderate |
| Li et al., 2021 | 8 | 7 | 7.5 | Moderate |
| Liu and Kang, 2021 | 7 | 7 | 7 | Moderate |
| Wang et al., 2021 | 8 | 8 | 8 | Low |
| Zhang et al., 2021 | 8 | 7 | 7.5 | Moderate |
| Zhang et al., 2021 | 8 | 8 | 8 | Low |
| He and Wu, 2022 | 6 | 5 | 5.5 | High |
| Jin et al., 2022 | 8 | 8 | 8 | Low |
| Tu et al., 2022 | 9 | 9 | 9 | Low |
| Zheng et al., 2022 | 8 | 8 | 8 | Low |
| Liu et al., 2023 | 7 | 7 | 7 | Moderate |
| Pan et al., 2023 | 8 | 8 | 8 | Low |
| Qin et al., 2023 | 7 | 8 | 7.5 | Moderate |
| Shan et al., 2023 | 8 | 9 | 8.5 | Low |
| Xu et al., 2023 | 8 | 8 | 8 | Low |
| You et al., 2023 | 6 | 6 | 6 | Moderate |
| Zhang et al., 2023 | 6 | 7 | 6.5 | Moderate |
| Yang et al., 2024 | 8 | 8 | 8 | Low |
| Yang et al., 2024 | 8 | 8 | 8 | Low |

**Appendix S6. Prevalence of syphilis among PLHIV via homosexual transmission.**

|  | Subgroup analysis | | | | |  | Univariate meta-regression | | | |
| --- | --- | --- | --- | --- | --- | --- | --- | --- | --- | --- |
|  | Number of studies | Number of individuals with syphilis coinfection | Number of PLHIV | Prevalence of syphilis coinfection (95% CI) | *I*^2^ (%) |  | β (SE) | OR (95% CI) | *P* value | *R*^2^ (%) |
| Region |  |  |  |  |  |  |  |  |  |  |
| Eastern region | 20 | 4238 | 15477 | 25.6% (20.8-31.2) | 98.1 |  | Ref | Ref | Ref | 5.95 |
| Central region | 2 | 124 | 325 | 38.2% (33.0-43.6) | 0.0 |  | 0.634 (0.455) | 1.884 (0.773-4.595) | 0.164 |  |
| Western region | 9 | 690 | 4080 | 19.7% (15.3-25.0) | 90.9 |  | -0.340 (0.243) | 0.712 (0.442-1.147) | 0.162 |  |
| Multiple regions | 2 | 812 | 2714 | 29.9% (28.2-31.7) | 0.0 |  | 0.210 (0.438) | 1.233 (0.523-2.910) | 0.632 |  |
| Year |  |  |  |  |  |  |  |  |  |  |
| Before 2010 | 10 | 2975 | 8482 | 33.8% (27.5-40.8) | 97.3 |  | Ref | Ref | Ref | 22.35 |
| 2010 or after | 23 | 2889 | 14114 | 21.4% (18.3-24.9) | 95.0 |  | -0.626 (0.206) | 0.535 (0.357-0.800) | 0.002 |  |
| Population |  |  |  |  |  |  |  |  |  |  |
| Community-based | 27 | 3460 | 14737 | 23.7% (19.6-28.2) | 97.1 |  | Ref | Ref | Ref | 2.15 |
| Hospital-based | 6 | 2404 | 7859 | 30.8% (22.7-40.3) | 98.3 |  | 0.361 (0.275) | 1.435 (0.838-2.459) | 0.188 |  |
| Risk of bias |  |  |  |  |  |  |  |  |  |  |
| High | 5 | 869 | 2968 | 27.9% (16.4-43.3) | 98.3 |  | Ref | Ref | Ref | <0.01 |
| Moderate | 12 | 3254 | 12909 | 22.4% (16.3-29.9) | 98.8 |  | -0.294 (0.332) | 0.745 (0.389-1.428) | 0.375 |  |
| Low | 16 | 1741 | 6719 | 25.8% (22.4-29.5) | 88.9 |  | -0.106 (0.321) | 0.900 (0.480-1.688) | 0.742 |  |
| Sample size |  |  |  |  |  |  |  |  |  |  |
| <1000 patients | 18 | 1825 | 6778 | 25.8% (20.0-32.6) | 97.0 |  | Ref | Ref | Ref | <0.01 |
| ≥1000 patients | 15 | 4039 | 15818 | 23.8% (19.2-29.2) | 98.0 |  | -0.110 (0.218) | 0.896 (0.584-1.375) | 0.616 |  |
| Syphilis testing method |  |  |  |  |  |  |  |  |  |  |
| Probable current syphilis infection | 25 | 3983 | 16056 | 23.6 (20.0-27.6) | 96.7 |  | Ref | Ref | Ref | 14.50 |
| Possible current infection, unspecified | 2 | 1132 | 2731 | 36.4 (25.2-49.4) | 92.5 |  | 0.610 (0.417) | 1.840 (0.813-4.166) | 0.143 |  |
| Lifetime syphilis infection | 4 | 349 | 1023 | 34.9 (31.1-39.0) | 30.7 |  | 0.613 (0.315) | 1.845 (0.996-3.419) | 0.052 |  |
| Unclear infection type | 2 | 400 | 2786 | 14.6 (12.7-16.8) | 50.1 |  | -0.571 (0.415) | 0.565 (0.251-1.273) | 0.168 |  |
| Total | 33 | 5864 | 22596 | 24.9% (21.3-28.9) | 97.5 |  |  |  |  |  |

Abbreviations: CI, confidence interval; OR, odds ratio; PLHIV, people living with HIV; Ref, reference category; SE, standard error.

**Appendix S7. Prevalence of syphilis among PLHIV via the heterosexual transmission route.**

|  | Subgroup analysis | | | | |  | Univariate meta-regression | | | |
| --- | --- | --- | --- | --- | --- | --- | --- | --- | --- | --- |
|  | Number of studies | Number of individuals with syphilis coinfection | Number of PLHIV | Prevalence of syphilis coinfection (95% CI) | *I*^2^ (%) |  | β (SE) | OR (95% CI) | *P* value | *R*^2^ (%) |
| Region |  |  |  |  |  |  |  |  |  |  |
| Eastern region | 8 | 878 | 5281 | 17.4% (11.6-25.2) | 97.3 |  | Ref | Ref | Ref | 16.60 |
| Central region | 2 | 239 | 976 | 24.8% (21.2-28.8) | 43.7 |  | 0.468 (0.520) | 1.597 (0.576-4.427) | 0.368 |  |
| Western region | 7 | 1301 | 15843 | 10.7% (6.1-18.0) | 99.0 |  | -0.570 (0.342) | 0.566 (0.289-1.106) | 0.096 |  |
| Year |  |  |  |  |  |  |  |  |  |  |
| Before 2010 | 3 | 436 | 1735 | 20.9% (12.3-33.2) | 96.1 |  | Ref | Ref | Ref | 1.40 |
| 2010 or after | 14 | 1982 | 20365 | 13.9% (9.7-19.6) | 98.6 |  | -0.474 (0.458) | 0.622 (0.253-1.529) | 0.301 |  |
| Population |  |  |  |  |  |  |  |  |  |  |
| Community-based | 11 | 1801 | 19079 | 12.6% (8.1-18.9) | 98.9 |  | Ref | Ref | Ref | 10.40 |
| Hospital-based | 6 | 617 | 3021 | 20.4% (13.6-29.4) | 95.9 |  | 0.577 (0.349) | 1.780 (0.899-3.526) | 0.098 |  |
| Risk of bias |  |  |  |  |  |  |  |  |  |  |
| High | 1 | 25 | 109 | 22.9% (16.0-31.7) | NA |  | Ref | Ref | Ref | <0.01 |
| Moderate | 5 | 1037 | 13180 | 12.4% (5.6-25.3) | 99.4 |  | -0.748 (0.834) | 0.474 (0.092-2.427) | 0.370 |  |
| Low | 11 | 1356 | 8811 | 15.7% (11.6-20.9) | 96.9 |  | -0.472 (0.798) | 0.624 (0.131-2.979) | 0.554 |  |
| Sample size |  |  |  |  |  |  |  |  |  |  |
| <1000 patients | 4 | 290 | 1504 | 20.4% (13.8-29.2) | 92.0 |  | Ref | Ref | Ref | 3.13 |
| ≥1000 patients | 13 | 2128 | 20596 | 13.5% (9.0-19.9) | 98.9 |  | -0.497 (0.407) | 0.608 (0.274-1.349) | 0.221 |  |
| Syphilis testing method |  |  |  |  |  |  |  |  |  |  |
| Probable current syphilis infection | 9 | 1228 | 11862 | 13.5 (8.5-20.8) | 98.4 |  | Ref | Ref | Ref | 39.26 |
| Possible current infection, unspecified | 2 | 420 | 1589 | 26.6 (15.7-41.3) | 97.1 |  | 0.843 (0.438) | 2.324 (0.985-5.485) | 0.054 |  |
| Lifetime syphilis infection | 4 | 380 | 1796 | 21.2 (16.9-26.3) | 81.3 |  | 0.539 (0.343) | 1.714 (0.876-3.354) | 0.116 |  |
| Unclear infection type | 2 | 390 | 6853 | 5.7 (5.2-6.3) | 0.0 |  | -0.915 (0.439) | 0.401 (0.169-0.947) | 0.037 |  |
| Total | 17 | 2418 | 22100 | 14.9% (10.6-20.6) | 98.7 |  |  |  |  |  |

Abbreviations: CI, confidence interval; OR, odds ratio; PLHIV, people living with HIV; Ref, reference category; SE, standard error.

**Appendix S8. Prevalence of syphilis among PLHIV via intravenous drug use.**

|  | Subgroup analysis | | | | |  | Univariate meta-regression | | | |
| --- | --- | --- | --- | --- | --- | --- | --- | --- | --- | --- |
|  | Number of studies | Number of individuals with syphilis coinfection | Number of PLHIV | Prevalence of syphilis coinfection (95% CI) | *I*^2^ (%) |  | β (SE) | OR (95% CI) | *P* value | *R*^2^ (%) |
| Region |  |  |  |  |  |  |  |  |  |  |
| Eastern region | 3 | 111 | 639 | 15.5% (7.7-28.8) | 90.2 |  | Ref | Ref | Ref | <0.01 |
| Central region | 2 | 15 | 93 | 16.1% (8.6-28.0) | 30.8 |  | 0.025 (1.316) | 1.026 (0.078-13.516) | 0.985 |  |
| Western region | 9 | 114 | 1670 | 9.9% (4.6-19.9) | 86.8 |  | -0.490 (0.963) | 0.612 (0.093-4.046) | 0.611 |  |
| Year |  |  |  |  |  |  |  |  |  |  |
| Before 2010 | 2 | 143 | 1000 | 14.4% (11.8-17.5) | 41.0 |  | Ref | Ref | Ref | <0.01 |
| 2010 or after | 12 | 97 | 1402 | 11.0% (5.6-20.4) | 87.5 |  | -0.345 (1.018) | 0.709 (0.096-5.210) | 0.735 |  |
| Population |  |  |  |  |  |  |  |  |  |  |
| Community-based | 13 | 238 | 2396 | 11.8% (7.8-17.6) | 86.8 |  | Ref | Ref | Ref | <0.01 |
| Hospital-based | 1 | 2 | 6 | 33.3% (8.4-73.2) | NA |  | 1.439 (1.556) | 4.217 (0.200-89.074) | 0.355 |  |
| Risk of bias |  |  |  |  |  |  |  |  |  |  |
| Moderate | 4 | 93 | 1437 | 6.1% (0.9-31.1) | 94.3 |  | Ref | Ref | Ref | <0.01 |
| Low | 10 | 147 | 965 | 13.5% (9.1-19.5) | 75.0 |  | -0.639 (0.858) | 0.528 (0.098-2.837) | 0.456 |  |
| Sample size |  |  |  |  |  |  |  |  |  |  |
| <1000 patients | 4 | 88 | 729 | 11.2% (6.0-19.9) | 58.6 |  | Ref | Ref | Ref | <0.01 |
| ≥1000 patients | 10 | 152 | 1673 | 12.2% (6.9-20.8) | 88.4 |  | -0.072 (0.836) | 0.931 (0.181-4.792) | 0.931 |  |
| Syphilis testing method |  |  |  |  |  |  |  |  |  |  |
| Probable current syphilis infection | 6 | 63 | 581 | 8.0 (3.0-19.5) | 89.8 |  | Ref | Ref | Ref | 38.64 |
| Possible current infection, unspecified | 1 | 65 | 405 | 16.0 (12.8-20.0) | NA |  | 0.770 (1.055) | 2.159 (0.273-17.075) | 0.466 |  |
| Lifetime syphilis infection | 6 | 112 | 754 | 21.0 (11.4-35.5) | 82.0 |  | 1.117 (0.614) | 3.055 (0.917-10.183) | 0.069 |  |
| Unclear infection type | 1 | 0 | 662 | 0.1 (0.0-1.2) | NA |  | -4.765 (1.760) | 0.009 (0.000-0.268) | 0.007 |  |
| Total | 14 | 240 | 2402 | 12.4% (8.3-18.3) | 85.9 |  |  |  |  |  |

Abbreviations: CI, confidence interval; OR, odds ratio; PLHIV, people living with HIV; Ref, reference category; SE, standard error.

**
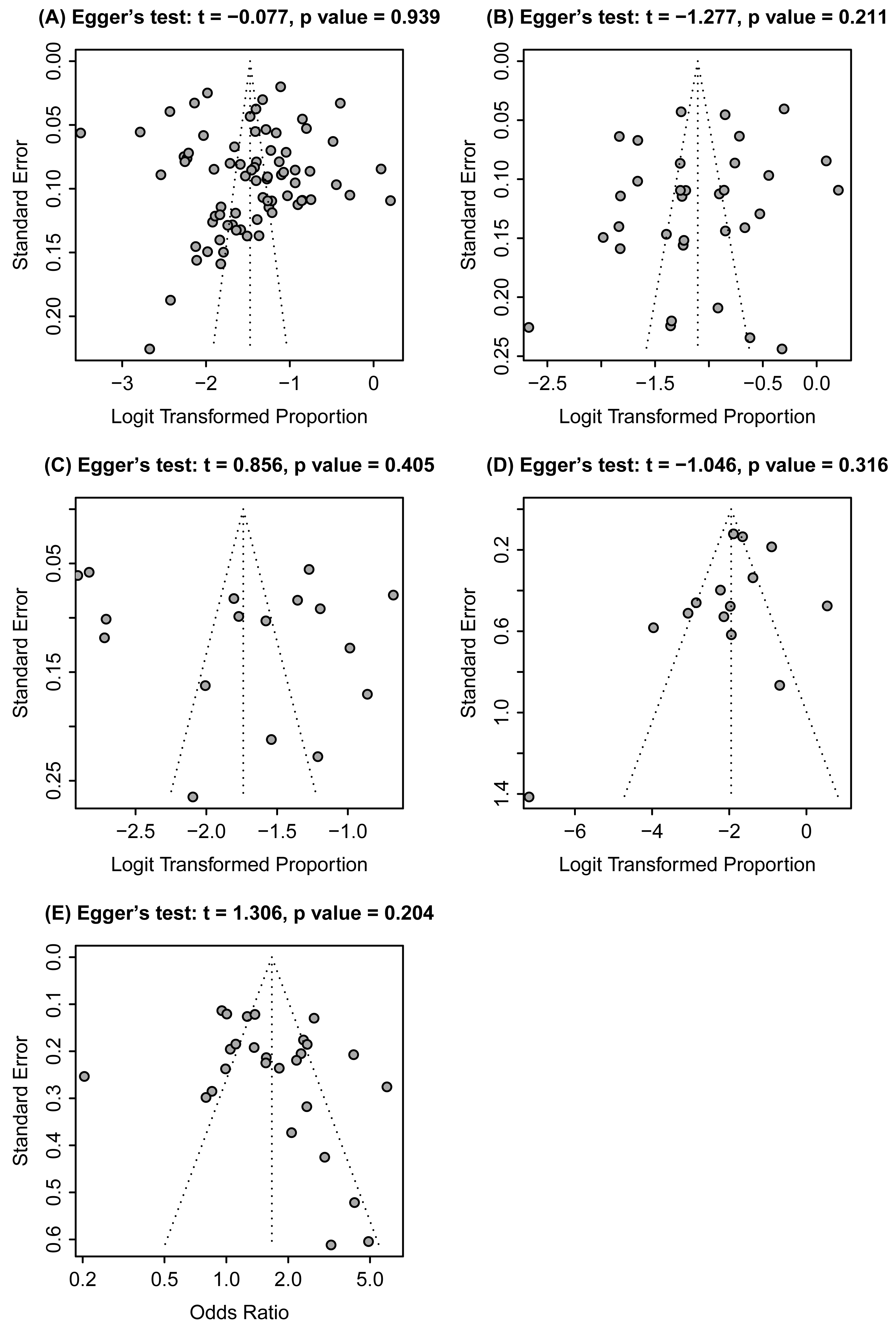
**

**Appendix Figure S1. Funnel plots and Egger’s tests for the outcomes: (A) overall, (B) homosexual transmission, (C) heterosexual transmission, (D) intravenous drug use, and (E) sex comparison.**
